# ICIsc: A Deep Learning Framework for Predicting Immune Checkpoint Inhibitor Response by Integrating scRNA-Seq and Protein Language Models

**DOI:** 10.3390/bioengineering13020187

**Published:** 2026-02-06

**Authors:** Zhenyu Jin, Di Zhang, Luonan Chen

**Affiliations:** 1 Key Laboratory of Systems Health Science of Zhejiang Province, School of Life Science, Hangzhou Institute for Advanced Study, University of Chinese Academy of Sciences, Chinese Academy of Sciences, Hangzhou 310024, China; jinzhenyu20@mails.ucas.ac.cn (Z.J.); zhangdi203@mails.ucas.ac.cn (D.Z.); 2 School of Mathematical Sciences and School of AI, Shanghai Jiao Tong University, Shanghai 200240, China

**Keywords:** deep learning, attention network, checkpoint inhibitors, immunotherapy response, single-cell RNA sequencing, single simple network

## Abstract

Immune checkpoint inhibitors (ICIs) targeting PD-1/PD-L1 and CTLA-4 are widely used in the treatment of several cancers and have significantly improved survival outcomes in responsive patients. However, a substantial proportion of patients fail to benefit from these therapies, underscoring the urgent need for accurate prediction of ICI response. We propose a deep learning framework, ICIsc, to accurately predict ICI response by integrating single-cell RNA sequencing (scRNA-seq) data with protein large language models. Specifically, patient representations are constructed using transcriptomic profiles and immune-related gene set scores as latent embedding features, while drug representations are derived from amino acid sequences of ICI encoded by the Evolutionary Scale Modeling 2 (ESM2). For bulk data, ICIsc employs a bilinear attention module to fuse patient and drug embeddings for response prediction. For scRNA-seq data, ICIsc infers cell–cell interactions using a single-sample network (SSN) approach and applies GATv2 to model immune microenvironment heterogeneity at the single-cell level. Benchmark evaluations and independent validation demonstrate that ICIsc consistently outperforms baseline models and exhibits robust generalization performance. SHAP-based interpretability analysis further identifies key genes (e.g., *GAPDH*) associated with immunotherapy response and patient prognosis. Overall, ICIsc provides an accurate and interpretable framework for predicting immunotherapy outcomes and elucidating underlying mechanisms.

## 1. Introduction

Cancer immunotherapy, particularly immune checkpoint blockade (ICI), has fundamentally transformed cancer treatment, leading to durable clinical responses in a subset of patients [1,2]. Among these approaches, blockade of the programmed cell death 1 (PD-1) receptor and its ligand PD-L1 represents one of the most widely exploited strategies by which cancer cells evade host antitumor immunity [3]. Another major immune checkpoint inhibitor, cytotoxic T-lymphocyte-associated antigen 4 (CTLA-4), plays a critical role in dampening T-cell activation and immune responses [4]. Despite the accelerated approval of multiple immunotherapeutic agents by the U.S. Food and Drug Administration (FDA), many patients do not respond to these treatments, making the identification of individuals most likely to benefit a pressing clinical challenge [5].

In recent studies, substantial progress has been made in characterizing survival outcomes and identifying prognostic biomarkers associated with ICI response, including cancer type, disease stage, microsatellite instability, and tumor mutational burden [6,7,8,9,10]. Existing studies often focus on specific cellular components within the tumor microenvironment (TME) [11,12]. However, the intricate and dynamic environment of immune interactions, together with the strong context dependency of immune responses, substantially limits the accuracy of current approaches for predicting patient-specific ICI efficacy. Currently, most immunotherapy response prediction models are predominantly based on conventional biomarkers. For example, Auslander et al. developed an immune predictive score based on pairwise transcriptional relationships among immune checkpoint genes to predict melanoma response to ICIs [13]. Jiang et al. proposed a model that quantifies Pearson correlations between patient transcriptomes and T-cell dysfunction signatures [14]. And Kong et al. transformed gene signatures into network-based representations by propagating ICI targets as hub genes through biological networks to identify pathways enriched with high-impact genes [15]. Similarly, Zhao et al. used ICI targets such as PD-1 or PD-L1 as anchor nodes to construct pathway networks enriched for target-related genes, which were subsequently incorporated into machine learning models to predict immunotherapy response [16].

Single-cell RNA sequencing (scRNA-seq) enables the characterization of cellular heterogeneity and dynamic cell states within the TME, providing a high-resolution view of immune responses at the individual cell level. As a result, scRNA-seq data hold substantial promise for improving the prediction of patient responses to ICIs. However, most existing approaches substantially simplify scRNA-seq data, leading to the loss of critical single-cell information and an inadequate representation of complex intercellular interactions within the TME [17,18,19]. For instance, Dong et al. trained machine learning models using cell-type proportions derived from scRNA-seq data to predict ICI efficacy, an approach that overlooks transcriptional diversity and functional interactions among immune cell populations [20].

In this study, we propose ICIsc, a deep learning framework for predicting patient responses to ICIs. ICIsc is designed to extract gene- and cell-level information from scRNA-seq data to improve immunotherapy response prediction. Compared with traditional machine learning approaches, ICIsc offers several key advantages. First, ICIsc introduces a truly multimodal and drug-aware framework for immunotherapy response prediction by jointly modeling patient transcriptomic profiles, immune pathway activities, and ICI antibody sequence embeddings. By incorporating drug-specific molecular representations, the model moves beyond the traditional paradigm that relies primarily on predefined immune checkpoint biomarkers, enabling the discovery of latent gene–drug–response interactions that are not captured by conventional approaches. Second, ICIsc establishes a unified cross-scale architecture capable of handling both bulk transcriptomic cohorts and scRNA-seq datasets, bridging two traditionally separate analytical levels and substantially expanding the framework’s applicability across experimental platforms. Third, through the integration of SSN-inferred cell–cell interaction features and a graph attention network v2 (GATv2), ICIsc explicitly models intercellular communication within the tumor microenvironment, capturing biologically meaningful heterogeneity beyond simple cell-type proportions. Finally, the incorporation of SHAP-based interpretability transforms the framework from a purely predictive model into a biologically informative system, enabling the identification of key genes and pathways linked to ICI response and prognosis while providing mechanistic insight into immunotherapy outcomes. These innovations collectively establish ICIsc as a framework that is more closely aligned with underlying biological principles, not only improving predictive performance but also deepening our understanding of the mechanisms driving immunotherapy response.

## 2. Materials and Methods

Our model, ICIsc, can be briefly described as follows. For bulk data, we collect patient gene expression profiles together with the antibody protein sequences of ICIs. Gene Set Variation Analysis (GSVA) is applied to derive pathway enrichment scores, which serve as patient-level features [21]. These features are encoded using multi-layer perceptrons, and a multi-head bilinear attention mechanism is subsequently employed to generate fused representations for final immunotherapy response prediction. For scRNA-seq data, following cell-type annotation, pseudo-bulk representations are constructed for each cell subset. Single-sample network (SSN) inference is then used to characterize interactions among cell subpopulations [22]. By integrating these interaction networks with cell subset proportions, a GATv2 is trained to predict patient responses to immunotherapy. We evaluated ICIsc across multiple immunotherapy clinical trial datasets and observed consistently superior performance compared with state-of-the-art ICI response prediction methods. Furthermore, independent and de novo validation analyses demonstrate that ICIsc exhibits robust generalization capability. Collectively, these results highlight the potential of the advanced computational framework ICIsc not only to enhance our understanding of cancer immunity but also to provide precision immunotherapy strategies.

### 2.1. Data Collection and Preprocessing

We systematically screened publicly available datasets related to cancer immunotherapy and collected pre-treatment transcriptomic profiles along with post-treatment response annotations from patients treated with ICIs, comprising a total of 957 samples, including both training and independent validation cohorts. Detailed information on the included datasets is provided in Table S1. All raw data were subjected to standardized preprocessing procedures. To mitigate batch effects across cohorts, we computed sample-level z-score normalization based on gene expression values, thereby enabling cohort-independent processing and facilitating the application of the framework to future individual patient data. Patients were subsequently classified as responders or non-responders according to the Response Evaluation Criteria in Solid Tumors (RECIST), as recent studies [14,15]. Specifically, patients exhibiting complete response or partial response were defined as responders, whereas those with stable disease or progressive disease were categorized as non-responders.

ScRNA-seq datasets were also retrieved from public repositories, including pre- and post-immunotherapy gene expression profiles together with corresponding response annotations for melanoma, triple-negative breast cancer, non-small-cell lung cancer, basal cell carcinoma, and breast cancer. In total, 211 samples were collected, comprising both training and independent validation cohorts. Detailed information on these datasets is provided in Table S1. Standard quality control, filtering, and preprocessing procedures were applied to all scRNA-seq data following established analytical pipelines. Cell subpopulations identified through unsupervised clustering were annotated using canonical marker genes in combination with the SingleR annotation framework [23]. Finally, gene expression values were averaged across cells within each annotated subpopulation to generate pseudo-bulk representations for downstream analyses.

### 2.2. Patient Feature Encoder

Let $S_{patient}=\left\{p_{1},p_{2},p_{3},...,p_{n}\right\}$ denote the set of all n patients. We compute latent representations of multi-omics data for cancer patients using a patient feature encoder (PFE). Specifically, we first derive patient feature vectors $a_{EXP}^{i}$ from gene expression profiles. To mitigate the adverse effects of scale differences among features during model training, z-score normalization is applied to the transcriptomic data. Given extensive prior research on the biological mechanisms underlying immunotherapy, we further enhance the biological interpretability of the model and facilitate the identification of potential mechanisms by incorporating collective gene behavior. To this end, immunotherapy-related gene sets are integrated to compute pathway enrichment scores, which are subsequently used as additional patient-level features. Specifically, based on prior work by the Li’s group, we curated gene sets associated with 371 ICI treatments [21]. These gene sets include markers related to the tumor immune microenvironment and immunotherapy response, with detailed descriptions provided in Table S3. We then employed the GSVA R package (version 3.20) to compute pathway enrichment scores. GSVA is a nonparametric and unsupervised approach that estimates sample-level enrichment scores for predefined gene sets. Its inputs include normalized gene expression profiles from the analyzed samples, gene sets representing biological pathways, and an optional kernel function. In this study, we used $a_{EXP}^{i}$ and the pathway gene set collection $S_{path}$ as inputs and specified a Gaussian kernel. GSVA first applies kernel density estimation to model the probability density of $a_{EXP}^{i}$, followed by transformation of these densities into normalized ranks to reduce noise. Kolmogorov–Smirnov (KS) random walk statistic relative to $p_{i}$ is computed for each gene set. The resulting normalized KS statistic is taken as the pathway enrichment score, denoted as $a_{PATH}^{i}$.

Next, we compute latent representations for $p_{i}$ using distinct multi-layer perceptrons (MLPs) based on $a_{EXP}^{i}$ and $a_{PATH}^{i}$. Within the PFE, each MLP consists of two hidden layers, with each layer in the MLP represented as follows:

(1) $$t_{mlp}^{i}=\sigma (\omega_{mlp}a_{mlp}^{i}+b_{mlp})$$

where $a^{i}$ represents the input data, $t^{i}$ represents the latent representation, ω and b denote learnable parameters, and σ denotes the rectified linear unit (ReLU) activation function. Ultimately, we obtain two latent representations of the same dimension for the patient: $t_{EXP}^{i}$ and $t_{PATH}^{i}$. These representations are then concatenated vertically into matrix $h_{p}^{i}\in R^{2\times l}$.

### 2.3. ICI Drug Feature Encoder

To derive ICI features, we retrieved antibody protein sequences corresponding to ICIs from DrugBank, including both light and heavy chains. These sequences were used as input to Evolutionary Scale Modeling 2 (ESM2), a deep learning model for protein sequence representation learning, to obtain latent embeddings of ICI drugs [24]. ESM2 employs a Transformer-based architecture to learn rich representations of protein sequences by capturing complex sequence patterns and evolutionary information, making it well-suited for downstream predictive tasks. In this study, we used the “esm2_t12_35M_UR50D” model with default parameters, resulting in 480-dimensional embeddings for each light or heavy chain.

### 2.4. Bilinear Attention Module

For bulk data, we employ a bilinear attention module to derive the final latent representation f for ICI drug-patient pairs. We first compute attention weights between patient and ICI drug latent representations. These drug–patient pair attention weights are then fed into a bilinear pooling layer. Taking a single-head bilinear attention network as an example, the first component is a bilinear attention layer that calculates interaction strengths between patient and ICI drug latent representations. For patient $p_{i}$ and drug $d_{j}$, we construct a bilinear attention graph using their latent representations $h_{p}^{j}$ and $h_{d}^{j}$ to obtain the interaction matrix I, expressed as:

(2) $$I=((1\cdot q^{T})\circ \sigma ({\left(h_{p}^{i}\right)}^{T}U))\cdot \sigma (V^{T}h_{d}^{j})$$

where U and V are learnable weight matrices, q is a learnable weight vector, 1 is the identity matrix, σ denotes the activation function, and ∘ represents the Hadamard product.

The second part is a bilinear pooling layer that processes the interaction matrix I to generate a fused representation $f^{\prime }$. The kth element of $f^{\prime }$ can be expressed as follows:

(3) $$f_{k}^{\prime }=\sigma {\left({\left(h_{p}^{i}\right)}^{T}U\right)}_{k}^{T}\cdot I\cdot \sigma {\left({\left(h_{d}^{i}\right)}^{T}V\right)}_{k}$$

The fused representation is ultimately fed into a pooling layer to obtain the final feature representation. Where SumPool(·) denotes a one-dimensional non-overlapping pooling operation with stride s=3.

### 2.5. Construction of Single-Sample Networks

We construct the state transition network by evaluating the statistical significance of all edges between samples while excluding the target single sample. For a given sample s, the corresponding network is constructed according to the following formulation:

(4) $$e_{ij}^{s}=N(e_{ij}^{N}-e_{ij}^{N-s})+e_{ij}^{N-s}$$

where $e_{ij}^{s}$ denotes the correlation between gene i and j in sample s, $e_{ij}^{N}$ represents the Pearson correlation coefficient between gene i and j in N samples, $e_{ij}^{N-s}$ indicates the PCC between gene i and j after removing sample s, and N denotes the total number of samples.

### 2.6. Graph Attention Network v2 Module

For single-cell RNA sequencing data, we employ GAT v2 to obtain the final latent representations of patients. Firstly, we obtain latent representations $h_{c}^{i}$ for each cell type as input node features. The interaction relationships between cell types i and j, derived from the SSN algorithm, serve as edge features. Here, H denotes the number of attention heads, and D represents the output dimension of each attention head. For each edge $\left(i,j\right)$, we compute the interaction between nodes i and j. Combining this with the edge feature E, we employ an additive attention mechanism, expressed as follows:

(5) $$a_{ij}=LeakyReLU(\partial^{T}\left[X_{i}∥X_{j}∥E_{ij}\right])$$

Here, ∂ denotes the learnable attention weight vector, $X_{i}$ and $X_{j}$ represent the latent projection features of nodes i and j, $E_{ij}$ denotes the edge feature, and ∥ indicates feature concatenation.

Next, we normalize the attention coefficients $a_{ij}$ using the softmax function to ensure the sum of each node’s neighbor weights equals 1. For each node i, the information received from neighboring node j is passed and updated through weighted attention coefficients, calculated as follows:

(6) $$h_{i}^{\prime }=\sum_{j}a_{ij}\left(X_{j}∥E_{ij}\right)$$

Finally, to prevent gradient vanishing in deep networks, we introduce residual connections. These add the node’s input features to its output features, generating the final node representation

### 2.7. Predictors

We employed a multi-layer perceptron (MLP) classifier to further transform the fused representations and generate the final prediction. The input layer of the MLP receives the fused feature vector. Different classifiers are adopted for the bulk and single-cell models, which are denoted as ${MLP}_{B}$ and ${MLP}_{S}$, respectively. The classifier consists of three fully connected hidden layers with 512 and 128 neurons, respectively, each followed by batch normalization (BN) and a Softmax activation function. Finally, a fully connected output layer with a single neuron produces the predicted probability of the response label.

### 2.8. Model Training

To minimize the discrepancy between predicted immunotherapy responses and ground-truth labels, we trained the model using the cross-entropy loss function, which is defined as follows:

(7) $$L=-\frac{1}{n}\sum_{i=1}^{n}\left[Y_{1}^{\left(i\right)}\log Y_{2}^{\left(i\right)}+(1-Y_{1}^{\left(i\right)})\log\left(1-Y_{2}^{\left(i\right)}\right)\right]$$

where n denotes the number of samples, $Y_{1}$ represents the true value, and $Y_{2}$ represents the predicted value. Additionally, several key hyperparameters were incorporated into the model, including the learning rate, batch size, number of attention heads, dropout rate, and the regularization parameter. These hyperparameters were optimized using a grid search strategy. In the grid search, the learning rate was varied across $\left\{1\times 10^{-2},1\times 10^{-3},1\times 10^{-4},1\times 10^{-5}\right\}$, the batch size across $\left\{32,64,128,256\right\}$, the number of attention heads across $\left\{1,2,3,4\right\}$, and the dropout rate across $\left\{0.1,0.2,0.3,0.4,0.5\right\}$. During hyperparameter tuning, we first optimized the learning rate and batch size. After fixing these values, we subsequently selected the number of attention heads and the dropout rate to further improve model performance and robustness. The final hyperparameter configurations are reported in the code repository.

## 3. Results

### 3.1. ICIsc Framework Overview

**Figure 1:**
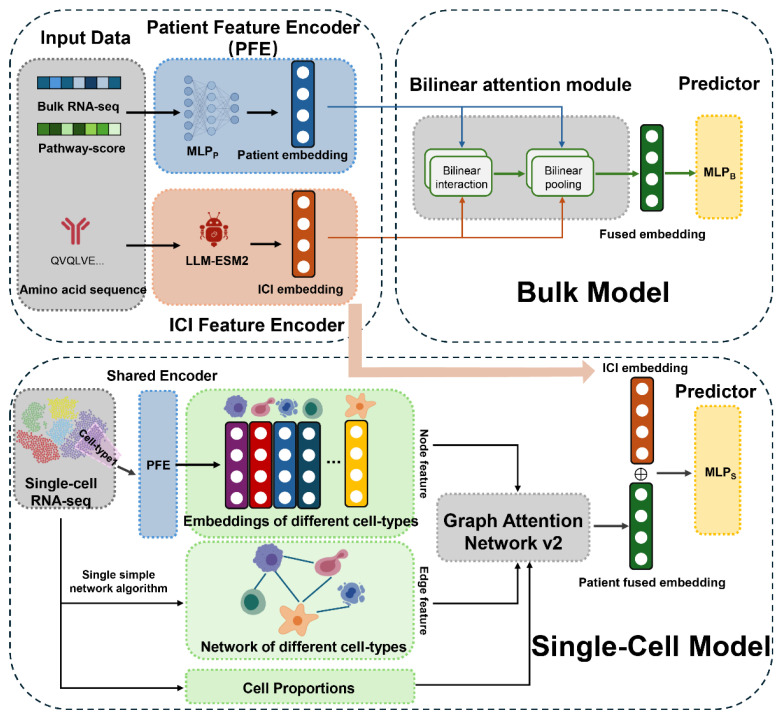
Overview of ICIsc framework. The model comprises three components. A patient feature encoder based on multi-layer perceptrons ${MLP}_{P}$ learns latent patient representations, while an ICI feature encoder employs the protein language model ESM2 to encode ICI drugs. In the bulk model, patient and ICI representations are integrated via a bilinear attention module to generate fused embeddings for response prediction using ${MLP}_{B}$. In the single-cell model, cell subtype embeddings, cell–cell interactions inferred by a single-sample network, and cell subtype proportions are incorporated into a GATv2 to derive patient representations, which are jointly used with ICI representations for immunotherapy response prediction using ${MLP}_{S}$.

In this study, we propose the ICIsc framework and workflow as shown in Figure 1. To comprehensively capture the influence of diverse genes on immunotherapy response, we integrated three types of data: (i) pre-treatment bulk transcriptomic or scRNA-seq data from patients enrolled in ICI clinical trials; (ii) pathway activity scores derived from immunotherapy-related gene sets; and (iii) antibody protein sequences of the ICIs administered. During model training, all three data modalities were jointly utilized, enabling the framework to embed potential gene- and pathway-level relationships relevant to immunotherapy response within the model architecture.

The ICIsc architecture consists of two main components: a bulk model and a single-cell model, both of which share a common patient feature encoder (PFE) and an ICI antibody protein sequence large language model. For the bulk model, we proceed as follows. First, based on the antibody protein sequences of the administered ICI drugs, we extract latent representations of the light and heavy chains using ESM2, a widely used deep learning model for protein sequences. The resulting representations are concatenated to form a unified latent representation of each ICI antibody drug with a dimensionality of 960. Second, GSVA is applied to patient gene expression profiles to compute pathway activity scores corresponding to immunotherapy-related gene sets. Third, gene expression features and pathway enrichment scores are jointly integrated into the cancer feature encoder. Within this encoder, feature-specific multi-layer perceptrons (${MLP}_{P}$) are designed to capture latent transcriptional patterns associated with cancer patients. Fourth, t these latent representations are fed into a bilinear attention module, which consists of a bilinear attention layer and a bilinear pooling layer, to generate fused representations of cancer patients and ICIs. Finally, these fused representations are passed to a ${MLP}_{B}$ to predict patient responses to immunotherapy. For the single-cell model, standard quality control, filtering, and preprocessing steps are first applied to the scRNA-seq data following established analysis pipelines. Cell subpopulations are identified via unsupervised clustering and subsequently annotated using canonical marker genes and the SingleR package, yielding the proportions of different cell subsets within each sample. And gene expression profiles are averaged within each cell subpopulation to construct pseudo-bulk representations. Following the same preprocessing strategy as in the bulk model, latent features are extracted for each cell subpopulation. Next, the SSN algorithm is employed to identify interaction relationships among cell subpopulations within the tumor microenvironment. These latent cell-subpopulation features are then used as node attributes, the inferred intercellular interactions as edge features, and the corresponding cell-type proportions as auxiliary inputs to a graph attention network. This network learns patient-level latent representations, which are finally input into a multi-layer perceptron to predict immunotherapy responses.

### 3.2. Evaluation of ICIs Model Performance on the Bulk Cohort

To evaluate the performance of ICIsc, we conducted a comprehensive comparative analysis using accuracy, F1 score, and the area under the receiver operating characteristic curve (AUROC) as evaluation metrics to assess its ability to predict immunotherapy response. ICIsc was benchmarked against five classical machine learning models (Support vector machine (SVM); Random forest (RF); Extreme gradient boosting (XGBoost); Elastic Net Regression (EN); Light Gradient Boosting Machine (LightGBM)) and two representative deep learning-based methods, IRnet [25]; TIDE [14] and iMLGAM [26]. All models were trained on benchmark datasets using either default or optimized hyperparameters, as appropriate. Each baseline method applied data preprocessing procedures consistent with its respective input features and methodological requirements. For the immunotherapy clinical trial datasets, samples were partitioned into five folds with comparable responder to non-responder ratios, and five-fold cross-validation was performed to ensure robust and unbiased performance evaluation. Detailed information on the immunotherapy clinical trial datasets is provided in Table S2.

**Figure 2:**
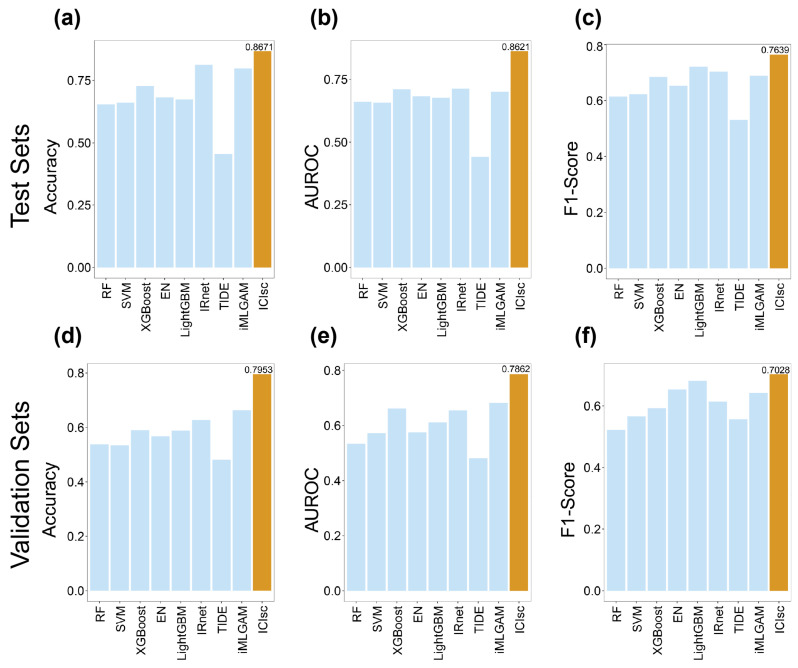
The performance of ICIsc in predicting immune checkpoint inhibitor (ICI) response across different Bulk RNA-seq immunotherapy datasets. (**a**–**c**) Bar chart showing the performance of different models on the test sets. From left to right, the metrics shown are (**a**) accuracy, (**b**) AUROC, and (**c**) F1-score. (**d**–**f**) Bar chart showing the performance of different models on the independent validation sets. From left to right, the metrics shown are (**d**) accuracy, (**e**) AUROC, and (**f**) F1-score.

Comparative performance results are summarized in Figure 2. Across all evaluation metrics, ICIsc consistently outperformed all baseline models. Specifically, ICIsc achieved an accuracy of 0.8671 (95%CI 0.7879–0.8932), an AUROC of 0.8621 (95%CI 0.7822–0.8733), and an F1 score of 0.7639, exceeding the performance of the remaining methods (Figure 2a–c). This performance advantage is likely attributable to the integrated modeling of molecular features from ICI drugs, transcriptomic profiles, and prior knowledge relevant to immunotherapy response. To further assess the generalizability of ICIsc, we conducted cross-cohort evaluations using two independent datasets. The results of these experiments are presented in Figure 2d–f. These cross-cohort evaluations spanned multiple cancer types and diverse ICI treatment settings. Compared with baseline models, ICIsc exhibited only a modest reduction in performance under cross-cohort conditions, while maintaining robust predictive capability, with an accuracy of 0.7953 (95%CI 0.7411–0.8207), an AUROC of 0.7862 (95%CI 0.7448–0.8190), and an F1 score of 0.7028. The results of DeLong’s test for AUROC comparison and McNemar’s test for accuracy comparison are presented in Tables S4 and S5. Collectively, these findings indicate that ICIsc demonstrates strong generalization ability, enabling reliable prediction of immunotherapy responses across heterogeneous cancer types and therapeutic agents.

To investigate the contributions of individual modules and feature components within the ICIsc framework, we performed a series of ablation experiments by systematically modifying model inputs and feature fusion strategies to assess the optimality of the proposed architecture and feature selection. Specifically, we designed several ICIsc variant models, including a version that uses only transcriptomic data as patient features and another that excludes ICI drug features while replacing the bilinear attention mechanism with a conventional attention module. The performance of these variant models is summarized in Table S8. Compared with the full ICIsc model, all variants exhibited varying degrees of performance degradation, with the most pronounced declines observed in models that excluded prior knowledge related to immunotherapy response. Notably, despite these reductions, all variant models still outperformed other baseline methods. These results demonstrate that each feature component contributes meaningfully to overall model performance and highlight the importance of integrating heterogeneous biological and drug-related features to achieve optimal predictive accuracy.

### 3.3. SHAP Values Reveal Key Feature Genes and Pathways

To identify key genes and immune pathways that are critical for predicting patient responses to ICI therapy and may serve as potential biomarkers, while accounting for complex nonlinear relationships among genes, we employed SHapley Additive exPlanations (SHAP) [27]. Derived from cooperative game theory, SHAP values quantify both the direction and magnitude of each gene’s contribution to the predicted immunotherapy response, enabling intuitive visualization of how gene expression influences model predictions. Importantly, SHAP captures context-dependent feature contributions; thus, even when two patients exhibit similar expression levels of a given gene, their SHAP values may differ depending on the broader transcriptional context.

**Figure 3:**
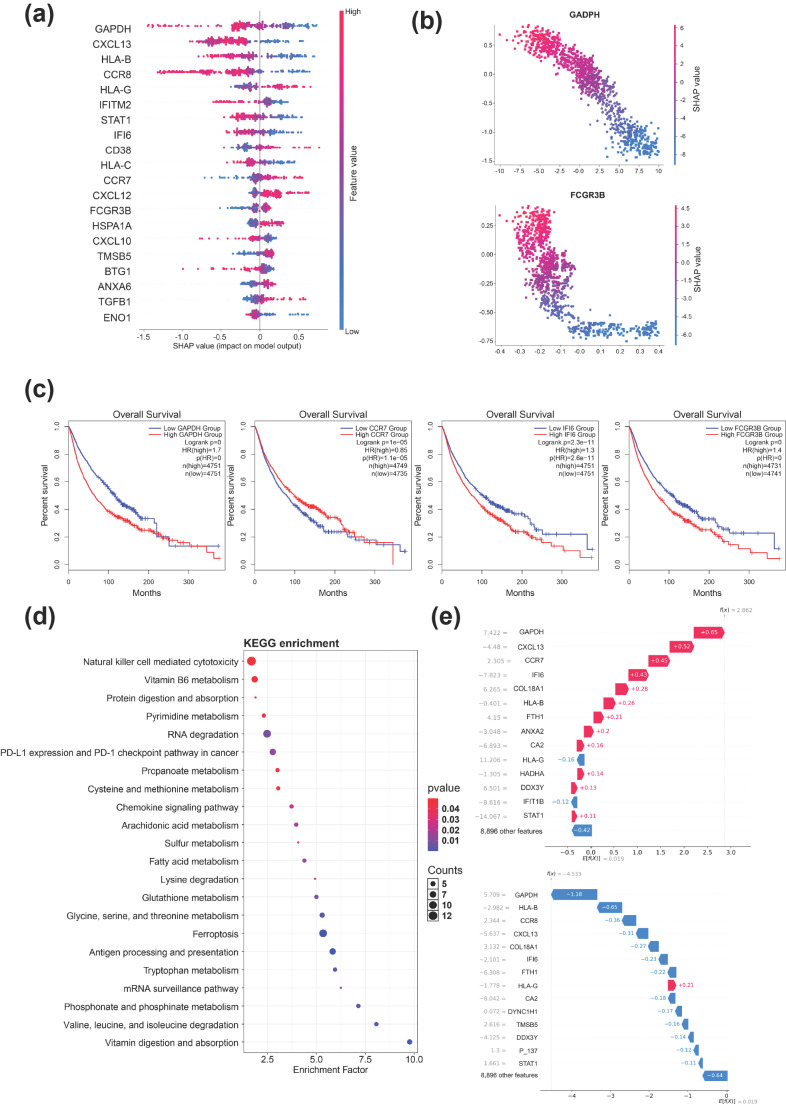
SHAP analysis of important features in immune response prediction. (**a**) SHAP value summary plot depicting the impact of each of the most important genes on model output. Gene expression (shown by coloring) as a function of SHAP value (*x*-axis) showing the relation between expression pattern and the model’s prediction. (**b**) Scatter plots showing the relationship between gene expression and SHAP values for key genes (*GAPDH* and *FCGR3B*). (**c**) Kaplan–Meier survival analysis of the 4 genes in the signature using bulk RNA-seq data across various cancer types. The survival curves illustrate the association between high and low expression of each gene. Plots include survival for genes—*GAPDH*, *CCR7*, *IFI6*, *FCGR3B*. (**d**) Dot plot showing differential KEGG pathway enrichment derived from the top 200 features. (**e**) Waterfall plots showing SHAP value contributions to individual model predictions.

From each fold of the five-fold cross-validation, we extracted the gene or immune pathway with the highest feature importance score. By analyzing the intersection of selected genes across all folds, we identified 10 genes that were consistently ranked as important in every fold: *GAPDH*, *CXCL13*, *IFI6*, *CCR7*, *FCGR3B*, *IFITM2*, *HLA-B*, *CD38*, *CXCL10*, and *STAT1* (Figure 3a). Notably, all of these genes have been previously implicated in tumor immunity and responses to ICI therapy. For example, *CD38*, a canonical macrophage marker, has been reported in single-cell expression analyses across multiple cancer types to be positively associated with favorable clinical outcomes [28,29,30]. Moreover, *CD38* has also been suggested as a potential biomarker for predicting the efficacy of PD-1 blockade immunotherapy [31]. Although *CD38* is commonly linked to M1-macrophages, which are generally considered to promote antitumor immunity, its prognostic role appears to be context-dependent within the tumor microenvironment [32]. *STAT1* has been reported to enhance immunotherapy responses in melanoma and to be positively correlated with PD-L1 expression in ovarian cancer [33,34]. In addition, *FCGR3B* is a cell-type-specific marker of NK cells and is considered to be involved in antibody-dependent cellular cytotoxicity. Previous studies have shown that NK cell-mediated cytotoxicity preferentially targets cells highly expressing PD-L1, suggesting an important role for antibody-dependent cellular cytotoxicity in enhancing immunotherapy efficacy, particularly in PD-L1-targeted immune checkpoint blockade [35,36,37]. Among all identified features, *GAPDH* exhibited the highest feature importance score and has been implicated in cancer progression and immune regulation, particularly through its involvement in hypoxia-associated pathways [38,39]. To further quantify the contributions of individual genes to model predictions, we computed SHAP values for these important features. As illustrated in Figure 3b, the directionality of SHAP values indicates that *GAPDH* is predominantly associated with non-response, with lower SHAP values corresponding to non-responders and higher SHAP values to responders. In contrast, *FCGR3B* displays a more threshold-like pattern, in which its expression levels more clearly discriminate responders from non-responders.

Finally, we investigated the association between the 10 identified feature genes and patient overall survival (OS) using a pan-cancer RNA sequencing dataset. Previous studies have shown that immune activation is broadly associated with improved survival outcomes across multiple cancer types. Based on this evidence, we hypothesized that ICIs efficacy is positively correlated with patient survival. To test this hypothesis, we used the GEPIA2 online platform to evaluate the relationship between expression levels of the 10 genes and OS across all available cancer types [40]. Using a median expression cutoff, we observed that 8 of the 10 genes (*GAPDH*, *CXCL13*, *IFI6*, *CCR7*, *FCGR3B*, *IFITM2*, *CXCL10*, and *STAT1*) were significantly associated with overall survival in the expected direction. In contrast, the remaining two genes, *HLA-B* and *CD38*, did not show a statistically significant association with survival (Figure 3c and Figure S1). Notably, the observed survival signal was consistent across multiple cancer types, and patients whose tumors exhibited higher expression of the survival-associated genes demonstrated improved outcomes. These findings further support our hypothesis that the predictive signals captured by ICIsc are driven, at least in part, by immune-related activation mechanisms.

To further characterize biomarkers associated with immunotherapy efficacy, we performed additional biological analyses on the top 200 features ranked by SHAP values, including 6 immune pathway gene set features and 194 individual genes. To explore their biological relevance, we focused on two representative gene sets: P_090 (exhausted T-cell gene set, ranked 39th) and P_146 (M2 macrophage gene set, ranked 87th). Based on SHAP value directionality, P_090 exhibited a positive association with immunotherapy response, whereas P_146 showed a negative association. Within the tumor microenvironment, T-cell exhaustion is a critical mechanism of tumor immune evasion. Immune checkpoint inhibitors exert their therapeutic effects primarily by reversing T-cell exhaustion, thereby restoring effector function and enhancing antitumor immune responses [41,42,43]. In contrast, M2-macrophages promote immunosuppression by secreting inhibitory cytokines such as *IL-10* and *TGF-β* and by interacting with tumor and immune cells, leading to suppression of T-cell and NK cell activity [44,45,46]. Through these mechanisms, M2-macrophages contribute to tumor progression, metastasis, and therapeutic resistance, and may consequently reduce the efficacy of immune checkpoint blockade. Collectively, these findings indicate that the top-ranked pathway features identified by ICIsc effectively capture key immunological processes underlying differential responses to immunotherapy.

In addition, pathway enrichment analysis of the remaining 194 genes revealed significant enrichment in pathways related to amino acid metabolism, energy metabolism, cofactor and vitamin metabolism, and carbohydrate metabolism. Metabolic dysregulation is widely recognized as a hallmark of cancer, and accumulating evidence indicates that metabolic pathways play a central role in regulating immune cell function and shaping responses to immunotherapy [47,48]. Specific amino acids, such as tryptophan and arginine, are essential for immune cell proliferation, differentiation, and effector activity [49]. Perturbations in amino acid metabolism, such as changes in transporter expression and enzymatic activity, can impair immune responses and ultimately reduce the efficacy of immunotherapeutic interventions. Energy metabolism further provides the metabolic foundation required to sustain immune responses by supplying ATP necessary for immune cell activation and effector functions [49]. Beyond metabolism-related pathways, ferroptosis-associated pathways were also identified as significantly enriched. Ferroptosis is a regulated form of cell death that has emerged as an important modulator of tumor progression and immune responses [50]. Recent studies have shown that combining ICIs with ferroptosis inducers can enhance antitumor immunotherapy efficacy. To further investigate the impact of amino acid metabolism and ferroptosis pathways on immunotherapy efficacy, we performed GSVA to calculate pathway activity scores in responders and non-responders (Figure 2 and Figures S3). The results showed that ferroptosis pathway scores were significantly higher in responders, whereas amino acid metabolism pathway scores exhibited the opposite trend. These findings suggest that ferroptosis-related metabolic reprogramming may modulate tumor susceptibility to immune-mediated killing. Enhanced ferroptosis sensitivity has been shown to synergize with immune checkpoint blockade by promoting *IFN-γ*-dependent lipid peroxidation in tumor cells [51,52]. We further examined the expression of key genes within these pathways (Figure S4). For example, *SLC7A11*, a critical negative regulator of ferroptosis, can be downregulated in tumor cells by interferon-γ secreted from activated T cells following ICI treatment, thereby increasing tumor cell susceptibility to ferroptosis [51]. In contrast, *IDO1*, a classical tryptophan-catabolizing enzyme and key immunosuppressive molecule, has been widely reported to promote tryptophan depletion when highly expressed, ultimately suppressing T-cell function [53]. Collectively, these results are well supported by existing literature and further corroborate the biological relevance and predictive accuracy of the ICIsc framework.

To further elucidate the behavior of the ICIsc model, we leveraged SHAP values to dissect the factors contributing to individual predictions and to better understand the model’s decision-making process. We focused on correctly classified samples to investigate the features underlying accurate predictions. SHAP waterfall plots were used to provide local explanations for individual predictions by visualizing the magnitude and direction of each gene’s contribution (Figure 3d). Two representative cases were selected for illustration: one responder and one non-responder. Across these correctly predicted samples, both responders and non-responders exhibited prominent contributions from several key genes, including *GAPDH*, *IFI6*, and *CXCL13*, consistent with the global feature importance results. Collectively, these findings demonstrate that SHAP-based local interpretability facilitates the identification of biologically relevant features associated with immunotherapy response and provides additional insight into the mechanisms captured by the ICIsc framework.

### 3.4. Evaluation of ICIs Model Performance on the Single-Cell Cohort

**Figure 4:**
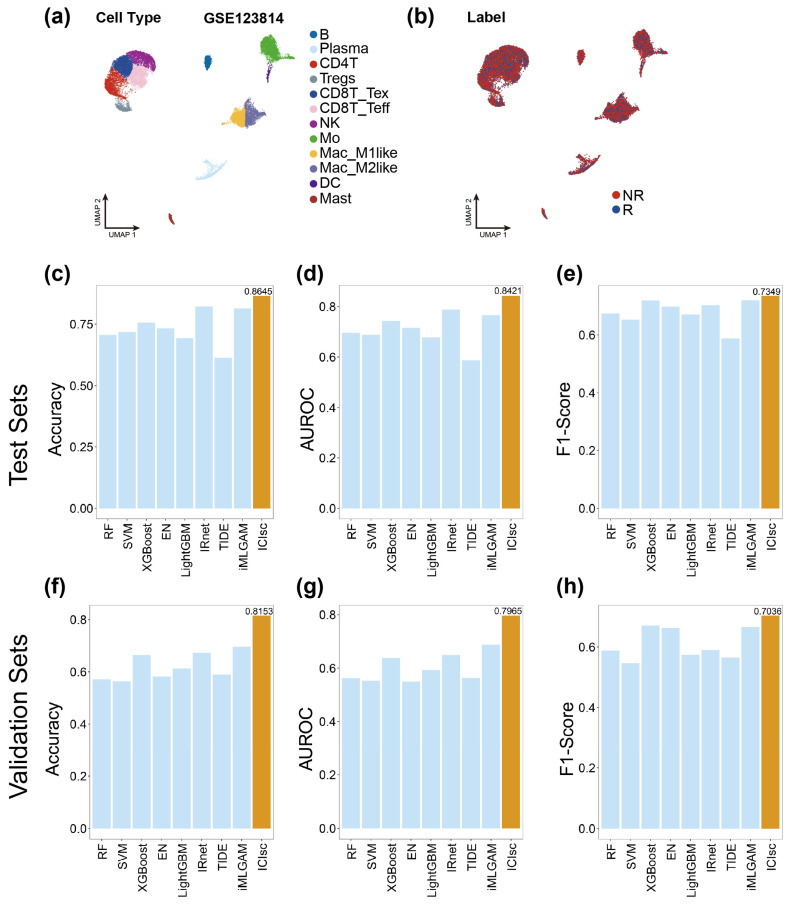
The performance of ICIsc in predicting immune checkpoint inhibitor (ICI) response across different scRNA-seq immunotherapy datasets. (**a**) UMAP plot of single immune cells of GSE123814, colored by disease subtypes. (**b**) UMAP plot of single immune cells of GSE123814, colored by truth label. (**c**–**e**) Bar chart showing the performance of different models on the test sets. From left to right, the metrics shown are (**c**) accuracy, (**d**) AUROC, and (**e**) F1-score. (**f**–**h**) Bar chart showing the performance of different models on the independent validationsets. From left to right, the metrics shown are (**f**) accuracy, (**g**) AUROC, and (**h**) F1-score.

Advances in scRNA-seq technologies have been instrumental in improving our understanding of the tumor immune microenvironment by enabling the characterization of cellular heterogeneity and the elucidation of complex intercellular interactions within tumors [43]. In the context of immune checkpoint therapy, treatment efficacy critically depends on coordinated interactions among diverse immune cell populations within the tumor microenvironment. Consequently, the analysis of scRNA-seq cohorts provides valuable insights into the mechanisms underlying heterogeneous immunotherapy responses. We subjected the scRNA-seq cohort with available immunotherapy response annotations to standard quality control, filtering, and preprocessing procedures. Cells were subsequently classified into 12 major cell types using canonical marker genes in combination with the SingleR package: B cells (B), plasma cells (Plasma), CD4+ T cells (CD4T), exhausted CD8+ T cells (CD8T_Tex), effector CD8+ T cells (CD8T_Teff), regulatory T cells (Tregs), natural killer (NK) cells, M1-like macrophages (Mac_M1like), M2-like macrophages (Mac_M2like), monocytes (Mo), dendritic cells (DC), and mast cells (Mast) (Figures S5 and S6). As detailed in the Table S10, the single-cell datasets encompass multiple cancer types, including basal cell carcinoma, triple-negative breast cancer, non–small cell lung cancer, melanoma, and classical breast cancer. Using the GSE123814 dataset as an illustrative example, after preprocessing, cell-type labels were evenly distributed, with an average of 1961 cells per patient (Figure 4a,b). In parallel, we computed patient-level node features by averaging gene expression profiles across each cell type, inferred intercellular interaction networks using the SSN algorithm to derive edge features, and calculated the relative proportions of each cell type as additional input features for downstream modeling.

To evaluate the performance of ICIsc on single-cell cohorts, we assessed its predictive accuracy using accuracy, F1 score, and AUROC as evaluation metrics. ICIsc was benchmarked against five classical machine learning methods and two representative deep learning approaches to assess its ability to predict immunotherapy responses from single-cell data. Owing to the inherent limitations of single-cell datasets, direct prediction of patient-level response labels is not feasible. Therefore, baseline models were designed to generate response predictions at the cell type level, and patient-level predictions were subsequently obtained by aggregating votes across cell-type-specific outputs. The model parameters and preprocessing procedures used for the baseline methods were largely consistent with those applied in the bulk transcriptomic analysis to ensure fair comparison. We employed five-fold cross-validation by partitioning the single-cell immunotherapy dataset into five folds, with each fold containing comparable proportions of responders and non-responders. In addition, the partitioning strategy ensured that each fold included samples from different cancer types, thereby reducing potential cohort-specific bias. Detailed information on the immunotherapy clinical trial datasets is provided in Table S2.

By integrating multi-dimensional features together with cell-type proportion information, ICIsc achieved superior predictive performance compared with all baseline models across all evaluation metrics. Comparative results are presented in Figure 4. Specifically, ICIsc attained an accuracy of 0.8645 (95%CI 0.8189–0.9195), an AUROC of 0.8421 (95%CI 0.8077–0.8926), and an F1 score of 0.7349, outperforming the competing methods (Figure 4c–e). We further assessed the generalizability of ICIsc using an independent single-cell breast cancer dataset. In this cross-cohort evaluation, ICIsc exhibited only a modest reduction in performance relative to the benchmark results, achieving an accuracy of 0.8153 (95%CI 0.7766–0.8461), an AUROC of 0.7965 (95%CI 0.7654–0.8335), and an F1 score of 0.7036, thereby maintaining robust predictive capability across datasets (Figure 4f–h). The results of DeLong’s test for AUROC comparison and McNemar’s test for accuracy comparison are presented in Tables S6 and S7. In addition, ablation experiments were conducted on the single-cell model to evaluate the contribution of individual components (Table S9). Variants of ICIsc that excluded ICI drug information or cell-type proportion features exhibited marked performance degradation. Similarly, substituting cell–cell interaction information derived from single-sample networks with conventional correlation-based measures led to reduced predictive performance. In addition, to further demonstrate the advantages of the SSN-based approach, we compared it with CellChat [54], a widely used method for constructing cell–cell communication networks in single-cell analyses. Communication strengths between different cell types inferred by CellChat were used to build alternative cell–cell interaction networks. However, models based on these networks still showed inferior performance compared with ICIsc. These results indicate that the proposed modules effectively capture immunotherapy response signals at a systems level. In summary, ICIsc demonstrates strong predictive performance on single-cell datasets and robust generalization ability across independent cohorts.

### 3.5. Analysis of Interactions Among Different Cell Types in Single-Cell Data

**Figure 5:**
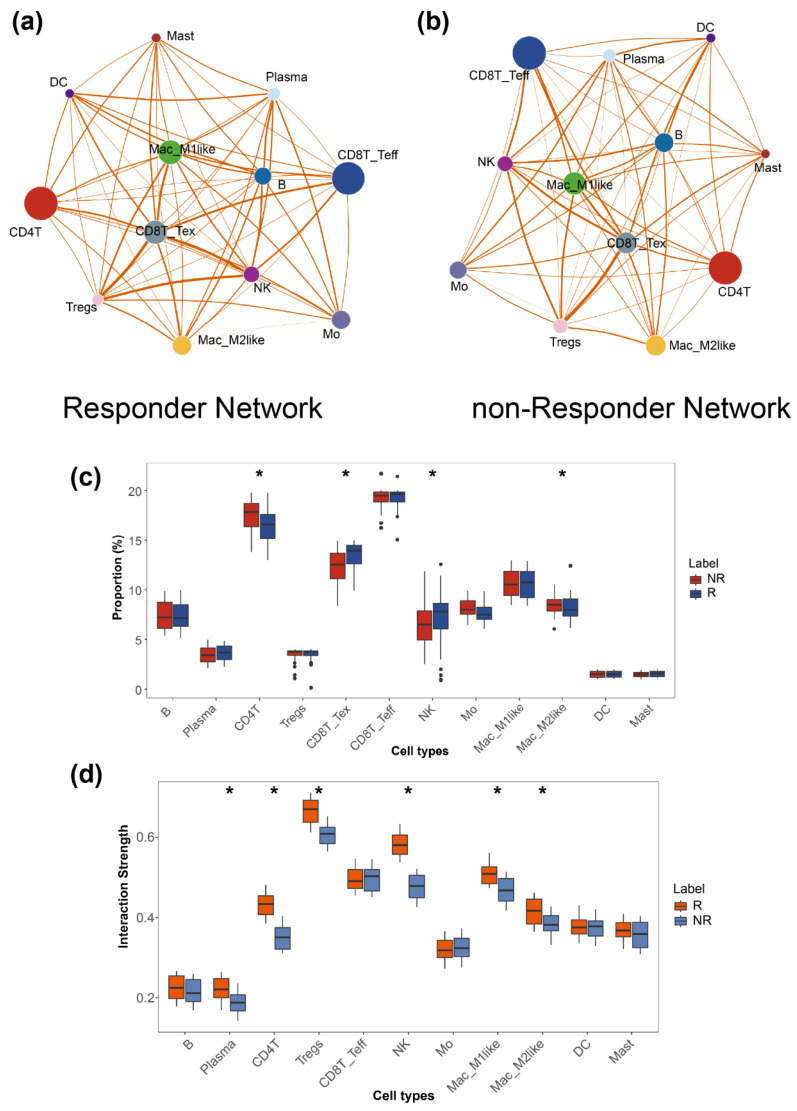
Cell–cell interactions across different cell types. (**a**,**b**) The interaction network diagram between different cell types, generated using the single simple network (SSN) algorithm. (**c**) Box plot showing the differences in the proportions of different cell subtypes in responders and non-responders. *p*-values were calculated by Wilcoxon test; * *p* < 0.05. (**d**) Box plot showing the differences in interaction strength between CD8T_tex and other cell types in responders and non-responders. *p*-values were calculated by Wilcoxon test; * *p* < 0.05.

The ICIsc model leverages a graph attention network and explicitly incorporates cell-type proportion information during training. Compared with traditional baseline models, this design enables the capture of interaction relationships among distinct cell types, which is critical for characterizing dynamic changes in the immune microenvironment during immunotherapy. To model cell–cell interactions, we employed the SSN inference approach. Unlike conventional network analysis methods, SSNs offer distinct advantages in identifying subject-specific features, potential biomarkers, and therapeutic targets [22]. Traditional network-based analyses may suffer from information loss or biased interpretations due to population-level heterogeneity. By focusing on individual samples, SSNs mitigate confounding effects arising from inter-group variability and more accurately reflect patient-specific biological states, while also enabling the detection of rare or subtle biological signals. As illustrated in Figure 5, we computed average cell–cell interaction networks for responders and non-responders. In these networks, node size represents the relative proportion of each cell type, while edge thickness denotes the strength of intercellular interactions (Figure 5a,b). Notably, responders exhibited stronger interaction intensities across most cell types compared with non-responders, with particularly pronounced interactions involving immunotherapy-relevant exhausted T cells and M2-macrophages. In addition, the proportions of both exhausted T cells and M2-macrophages were higher in responders than in non-responders (Figure 5c). Furthermore, quantitative analysis of interaction strengths between exhausted T cells or M2-macrophages and other immune cell populations revealed consistently higher interaction intensities in responders compared with non-responders (Figure 5d and Figure S7). These observations are consistent with prior studies demonstrating that immune checkpoint inhibitors can relieve immune suppression and reinvigorate T-cell activity, thereby restoring antitumor immune function [41]. Collectively, our findings highlight a close association between immunotherapy response and the coordinated behavior of exhausted T cells and M2-macrophages, in agreement with existing studies [14,32].

## 4. Discussion

This study introduces ICIsc, a deep learning framework for predicting the efficacy of ICI therapy. Unlike traditional approaches that rely on predefined marker genes, ICIsc integrates multimodal information from both cancer patients and ICI drugs. Specifically, it derives patient representations from transcriptomic profiles and immune pathway activity scores based on immune response-related gene sets, while leveraging the protein large language model ESM2 to encode drug-specific characteristics [24]. By utilizing genome-wide transcriptomic information, ICIsc enables the identification of potential biomarkers while reducing subjective bias introduced by manual feature selection. Importantly, ICIsc demonstrates robust predictive performance across both bulk and single-cell datasets. Extensive evaluations on multiple benchmark datasets and independent validation cohorts consistently show that ICIsc outperforms existing methods. Furthermore, feature importance analyses and ablation studies confirm the effectiveness and reliability of the proposed feature selection and model design for immunotherapy response prediction.

Beyond its strong predictive performance, ICIsc offers multi-level interpretability through SHAP-based analyses. By leveraging interpretable deep learning models, we identified potential biomarkers and biological pathways that influence responses to ICI therapy. Notably, hypoxia-related genes such as *GAPDH* were highlighted as key contributors and have been previously reported to correlate with immunotherapy outcomes [38,39]. In addition, pathways associated with metabolic processes and cell growth or death were found to be closely linked to immunotherapy response. Together, these results demonstrate that the interpretability of ICIsc enables the elucidation of biologically meaningful mechanisms underlying differential responses to immunotherapy, thereby increasing confidence in the model’s predictions and supporting its potential utility in translational and clinical research.

Despite these advances, current approaches remain subject to important limitations and substantial opportunities for improvement. The most critical challenge is the scarcity of large-scale, publicly available immunotherapy datasets. Existing datasets typically comprise only dozens of patients and are therefore vulnerable to batch effects during model training, arising from heterogeneity in cancer types, treatment regimens, and sequencing platforms. Previous studies have shown that, despite considerable investment in cancer immunotherapy research, many available datasets still suffer from limited usability and standardization. Expanding the collection of immunotherapy patient data would substantially improve the predictive accuracy and robustness of models such as ICIsc. Analysis of larger and more diverse patient cohorts will further advance precision medicine by enabling more reliable patient stratification. Moreover, increased data availability may facilitate the discovery of novel pathways and therapeutic targets associated with ICI response, thereby supporting downstream biological investigations and the development of next-generation immunotherapies. Meanwhile, large language models have demonstrated strong performance in traditional small-molecule drug discovery and prediction tasks [55,56]. The availability of large-scale, well-curated, and standardized datasets will also enable the development of domain-specific large language models tailored to immunotherapy research. Several existing studies have demonstrated that incorporating additional clinical or multi-omics information, such as tumor mutational burden (TMB) and radiomic features, may further improve the accuracy of immunotherapy response prediction models [57,58,59]. Moreover, compared with transcriptomic profiling, these data types are often more readily accessible in clinical settings. In future work, we plan to standardize a broader range of datasets and extend our current framework by integrating additional clinical and multi-omics variables. We also aim to explore predictive models for combination therapies involving ICIs and chemotherapeutic agents, with the goal of better reflecting real-world treatment strategies, optimizing combination regimens, and ultimately improving therapeutic outcomes.

## 5. Conclusions

In summary, we developed and further validated ICIsc, a deep learning framework that predicts the efficacy of ICI therapy by integrating scRNA-seq with protein-specific large language models. The proposed ICIsc model demonstrated strong robustness and stability in cross-cohort evaluations and provided interpretable feature-level explanations. Its key advantages lie in the joint integration of ICI drug-specific characteristics, the ability to uncover latent gene-level features, and the capability to capture heterogeneity within the immune microenvironment. Collectively, our framework holds significant value for advancing the understanding of cancer immunity and guiding the clinical use of ICI therapies.

## Data Availability

The data supporting the findings of this study are publicly available. Detailed information on data sources and accession numbers is provided in the Supplementary Materials. The source code for ICIsc has been made publicly available on GitHub: https://github.com/HIAS-chenlab/ICIsc (accessed on 1 February 2026).

## Supplementary Materials

The following supporting information can be downloaded at: https://www.mdpi.com/article/10.3390/bioengineering13020187/s1, Figure S1: Kaplan–Meier survival analysis of the 6 genes in the signature using bulk RNA-seq data across various cancer types. The survival curves illustrate the association between high and low expression of each gene. Plots include survival for genes—CXCL13, IFITM2, STAT1, CXCL10, HLA-B, CD38. Figure S2: Violin plot showing ferroptosis-related gene set scores in responders and non-responders. *p*-values were calculated by Wilcoxon test; * *p* < 0.05. Figure S3: Violin plot showing amino acid metabolism-related gene set scores in responders and non-responders. *p*-values were calculated by Wilcoxon test; * *p* < 0.05. Figure S4: Box plots showing the expression levels of selected feature genes from the ferroptosis and amino acid metabolism pathways in responders and non-responders. *p*-values were calculated by Wilcoxon test; * *p* < 0.05. Figure S5: Dot plot showing the expression of cell-type-specific marker. Figure S6: Fraction of immune cells originating from each patient in GSE123814. Figure S7: Box plot showing the differences in interaction strength between Mac_M2like and other cell types in responders and non-responders. *p*-values were calculated by Wilcoxon test; * *p* < 0.05. Table S1: Summary of datasets. Table S2: Summary of datasets. Table S3: Gene sets associated with the tumor microenvironment and immunotherapy. Table S4: Comparative Performance of ICIsc and Baseline Models in Bulk Test Cohorts with 95% Confidence Intervals and Statistical Significance Testing. Table S5: Comparative Performance of ICIsc and Baseline Models in Bulk Validation Cohorts with 95% Confidence Intervals and Statistical Significance Testing. Table S6: Comparative Performance of ICIsc and Baseline Models in Single-Cell Test Cohorts with 95% Confidence Intervals and Statistical Significance Testing. Table S7: Comparative Performance of ICIsc and Baseline Models in Single-Cell Validation Cohorts with 95% Confidence Intervals and Statistical Significance Testing. Table S8: Ablation experiment results of variant models in which one feature is removed for ICIsc–bulk model. Table S9: Ablation experiment results of variant models for ICIsc model. Table S10: Fraction of immune cells originating from each patient in scRNA-seq datasets.

## Abbreviations

ICI	Immune checkpoint inhibitor
scRNA-seq	Single-cell RNA sequencing
ESM2	Evolutionary Scale Modeling 2
SSN	Single-sample network
PD-1	Programmed cell death 1
CTLA-4	Cytotoxic T-lymphocyte-associated antigen 4
FDA	Food and Drug Administration
TME	Tumor microenvironment
SVM	Support vector machine
GATv2	Graph attention network v2
RF	Random forest
EN	Elastic Net Regression
LightGBM	Light Gradient Boosting Machine
XGBoost	Extreme gradient boosting
GSVA	Gene Set Variation Analysis
RECIST	Response Evaluation Criteria in Solid Tumors
KS	Kolmogorov–Smirnov
MLP	Multi-layer perceptron
ReLU	Rectified linear unit
AUROC	The area under the receiver operating characteristic curve
SHAP	SHapley Additive exPlanations
OS	Overall survival
PFE	Patient feature encoder

## References

[1] Pardoll D.M. (2012). The Blockade of Immune Checkpoints in Cancer Immunotherapy. Nat. Rev. Cancer.

[2] Sharma P., Hu-Lieskovan S., Wargo J.A., Ribas A. (2017). Primary, Adaptive, and Acquired Resistance to Cancer Immunotherapy. Cell.

[3] Nowicki T.S., Hu-Lieskovan S., Ribas A. (2018). Mechanisms of Resistance to PD-1 and PD-L1 Blockade. Cancer J..

[4] Ribas A., Wolchok J.D. (2018). Cancer Immunotherapy Using Checkpoint Blockade. Science.

[5] Sharma P., Goswami S., Raychaudhuri D., Siddiqui B.A., Singh P., Nagarajan A., Liu J., Subudhi S.K., Poon C., Gant K.L. (2023). Immune Checkpoint Therapy—Current Perspectives and Future Directions. Cell.

[6] Nixon N.A., Blais N., Ernst S., Kollmannsberger C., Bebb G., Butler M., Smylie M., Verma S. (2018). Current Landscape of Immunotherapy in the Treatment of Solid Tumours, with Future Opportunities and Challenges. Curr. Oncol..

[7] Gridelli C., Rossi A., Carbone D.P., Guarize J., Karachaliou N., Mok T., Petrella F., Spaggiari L., Rosell R. (2015). Non-Small-Cell Lung Cancer. Nat. Rev. Dis. Primers.

[8] Xu Y., Wan B., Chen X., Zhan P., Zhao Y., Zhang T., Liu H., Afzal M.Z., Dermime S., Hochwald S.N. (2019). The Association of PD-L1 Expression with the Efficacy of Anti-PD-1/PD-L1 Immunotherapy and Survival of Non-Small Cell Lung Cancer Patients: A Meta-Analysis of Randomized Controlled Trials. Transl. Lung Cancer Res..

[9] Strickler J.H., Hanks B.A., Khasraw M. (2021). Tumor Mutational Burden as a Predictor of Immunotherapy Response: Is More Always Better?. Clin. Cancer Res..

[10] Marabelle A., Fakih M., Lopez J., Shah M., Shapira-Frommer R., Nakagawa K., Chung H.C., Kindler H.L., Lopez-Martin J.A., Miller W.H. (2020). Association of Tumour Mutational Burden with Outcomes in Patients with Advanced Solid Tumours Treated with Pembrolizumab: Prospective Biomarker Analysis of the Multicohort, Open-Label, Phase 2 KEYNOTE-158 Study. Lancet Oncol..

[11] Wu T., Dai Y. (2017). Tumor Microenvironment and Therapeutic Response. Cancer Lett..

[12] Toor S.M., Sasidharan Nair V., Decock J., Elkord E. (2020). Immune Checkpoints in the Tumor Microenvironment. Semin. Cancer Biol..

[13] Auslander N., Zhang G., Lee J.S., Frederick D.T., Miao B., Moll T., Tian T., Wei Z., Madan S., Sullivan R.J. (2018). Robust Prediction of Response to Immune Checkpoint Blockade Therapy in Metastatic Melanoma. Nat. Med..

[14] Jiang P., Gu S., Pan D., Fu J., Sahu A., Hu X., Li Z., Traugh N., Bu X., Li B. (2018). Signatures of T Cell Dysfunction and Exclusion Predict Cancer Immunotherapy Response. Nat. Med..

[15] Kong J., Ha D., Lee J., Kim I., Park M., Im S.-H., Shin K., Kim S. (2022). Network-Based Machine Learning Approach to Predict Immunotherapy Response in Cancer Patients. Nat. Commun..

[16] Zhao L., Qi X., Chen Y., Qiao Y., Bu D., Wu Y., Luo Y., Wang S., Zhang R., Zhao Y. (2023). Biological Knowledge Graph-Guided Investigation of Immune Therapy Response in Cancer with Graph Neural Network. Brief. Bioinform..

[17] Kang Y., Vijay S., Gujral T.S. (2022). Deep Neural Network Modeling Identifies Biomarkers of Response to Immune-Checkpoint Therapy. iScience.

[18] Ma Q., Xu D. (2022). Deep Learning Shapes Single-Cell Data Analysis. Nat. Rev. Mol. Cell Biol..

[19] Erfanian N., Heydari A.A., Feriz A.M., Iañez P., Derakhshani A., Ghasemigol M., Farahpour M., Razavi S.M., Nasseri S., Safarpour H. (2023). Deep Learning Applications in Single-Cell Genomics and Transcriptomics Data Analysis. Biomed. Pharmacother..

[20] Dong Y., Chen Z., Yang F., Wei J., Huang J., Long X. (2024). Prediction of Immunotherapy Responsiveness in Melanoma through Single-Cell Sequencing-Based Characterization of the Tumor Immune Microenvironment. Transl. Oncol..

[21] Hänzelmann S., Castelo R., Guinney J. (2013). GSVA: Gene Set Variation Analysis for Microarray and RNA-Seq Data. BMC Bioinform..

[22] Huang Y., Chang X., Zhang Y., Chen L., Liu X. (2021). Disease Characterization Using a Partial Correlation-Based Sample-Specific Network. Brief. Bioinform..

[23] Aran D., Looney A.P., Liu L., Wu E., Fong V., Hsu A., Chak S., Naikawadi R.P., Wolters P.J., Abate A.R. (2019). Reference-Based Analysis of Lung Single-Cell Sequencing Reveals a Transitional Profibrotic Macrophage. Nat. Immunol..

[24] Lin Z., Akin H., Rao R., Hie B., Zhu Z., Lu W., Smetanin N., Verkuil R., Kabeli O., Shmueli Y. (2023). Evolutionary-Scale Prediction of Atomic Level Protein Structure with a Language Model. Science.

[25] Jiang Y., Immadi M.S., Wang D., Zeng S., On Chan Y., Zhou J., Xu D., Joshi T. (2025). IRnet: Immunotherapy Response Prediction Using Pathway Knowledge-Informed Graph Neural Network. J. Adv. Res..

[26] Ye B., Fan J., Xue L., Zhuang Y., Luo P., Jiang A., Xie J., Li Q., Liang X., Tan J. (2025). iMLGAM: Integrated Machine Learning and Genetic Algorithm-driven Multiomics Analysis for Pan-cancer Immunotherapy Response Prediction. iMeta.

[27] Lundberg S.M., Lee S.-I. (2017). A Unified Approach to Interpreting Model Predictions. NIPS’17: Proceedings of the 31st International Conference on Neural Information Processing Systems.

[28] Wu P., Zhao L., Chen Y., Xin Z., Lin M., Hao Z., Chen X., Chen D., Wu D., Chai Y. (2021). CD38 Identifies Pre-Activated CD8+ T Cells Which Can Be Reinvigorated by Anti-PD-1 Blockade in Human Lung Cancer. Cancer Immunol. Immunother..

[29] Lv L.-L., Zhai J.-W., Wu J.-J., Fan G.-Q., Zhang Y.-X., Shen Y., Qu Q.-X., Chen C. (2025). High CD38 Expression Defines a Mitochondrial Function-Adapted CD8+ T Cell Subset with Implications for Lung Cancer Immunotherapy. Cancer Immunol. Immunother..

[30] Ng H.H.M., Lee R.Y., Goh S., Tay I.S.Y., Lim X., Lee B., Chew V., Li H., Tan B., Lim S. (2020). Immunohistochemical Scoring of CD38 in the Tumor Microenvironment Predicts Responsiveness to Anti-PD-1/PD-L1 Immunotherapy in Hepatocellular Carcinoma. J. Immunother. Cancer.

[31] Chen L., Diao L., Yang Y., Yi X., Rodriguez B.L., Li Y., Villalobos P.A., Cascone T., Liu X., Tan L. (2018). CD38-Mediated Immunosuppression as a Mechanism of Tumor Cell Escape from PD-1/PD-L1 Blockade. Cancer Discov..

[32] Oshi M., Tokumaru Y., Asaoka M., Yan L., Satyananda V., Matsuyama R., Matsuhashi N., Futamura M., Ishikawa T., Yoshida K. (2020). M1 Macrophage and M1/M2 Ratio Defined by Transcriptomic Signatures Resemble Only Part of Their Conventional Clinical Characteristics in Breast Cancer. Sci. Rep..

[33] Liu F., Liu J., Zhang J., Shi J., Gui L., Xu G. (2020). Expression of STAT1 Is Positively Correlated with PD-L1 in Human Ovarian Cancer. Cancer Biol. Ther..

[34] Horowitch B., Lee D.Y., Ding M., Martinez-Morilla S., Aung T.N., Ouerghi F., Wang X., Wei W., Damsky W., Sznol M. (2023). Subsets of IFN Signaling Predict Response to Immune Checkpoint Blockade in Patients with Melanoma. Clin. Cancer Res..

[35] Meng F., Zhang S., Xie J., Zhou Y., Wu Q., Lu B., Zhou S., Zhao X., Li Y. (2023). Leveraging CD16 Fusion Receptors to Remodel the Immune Response for Enhancing Anti-Tumor Immunotherapy in iPSC-Derived NK Cells. J. Hematol. Oncol..

[36] Aguilar O.A., Gonzalez-Hinojosa M.D.R., Arakawa-Hoyt J.S., Millan A.J., Gotthardt D., Nabekura T., Lanier L.L. (2023). The CD16 and CD32b Fc-Gamma Receptors Regulate Antibody-Mediated Responses in Mouse Natural Killer Cells. J. Leukoc. Biol..

[37] Park J.-E., Kim S.-E., Keam B., Park H.-R., Kim S., Kim M., Kim T.M., Doh J., Kim D.-W., Heo D.S. (2020). Anti-Tumor Effects of NK Cells and Anti-PD-L1 Antibody with Antibody-Dependent Cellular Cytotoxicity in PD-L1-Positive Cancer Cell Lines. J. Immunother. Cancer.

[38] Peng C., Ye H., Yi Z. (2023). GAPDH: Unveiling Its Impact as a Key Hypoxia-Related Player in Head and Neck Squamous Cell Carcinoma Tumor Progression, Prognosis, and Therapeutic Potential. Am. J. Cancer Res..

[39] Wang J., Yu X., Cao X., Tan L., Jia B., Chen R., Li J. (2023). GAPDH: A Common Housekeeping Gene with an Oncogenic Role in Pan-Cancer. Comput. Struct. Biotechnol. J..

[40] Tang Z., Kang B., Li C., Chen T., Zhang Z. (2019). GEPIA2: An Enhanced Web Server for Large-Scale Expression Profiling and Interactive Analysis. Nucleic Acids Res..

[41] Liu B., Zhang Y., Wang D., Hu X., Zhang Z. (2022). Single-Cell Meta-Analyses Reveal Responses of Tumor-Reactive CXCL13+ T Cells to Immune-Checkpoint Blockade. Nat. Cancer.

[42] Chow A., Perica K., Klebanoff C.A., Wolchok J.D. (2022). Clinical Implications of T Cell Exhaustion for Cancer Immunotherapy. Nat. Rev. Clin. Oncol..

[43] Liu Z., Yang Z., Wu J., Zhang W., Sun Y., Zhang C., Bai G., Yang L., Fan H., Chen Y. (2025). A Single-Cell Atlas Reveals Immune Heterogeneity in Anti-PD-1-Treated Non-Small Cell Lung Cancer. Cell.

[44] Yamaguchi Y., Gibson J., Ou K., Lopez L.S., Ng R.H., Leggett N., Jonsson V.D., Zarif J.C., Lee P.P., Wang X. (2022). PD-L1 Blockade Restores CAR T Cell Activity through IFN-γ-Regulation of CD163+ M2 Macrophages. J. Immunother. Cancer.

[45] Wang D.-R., Wu X.-L., Sun Y.-L. (2022). Therapeutic Targets and Biomarkers of Tumor Immunotherapy: Response versus Non-Response. Signal Transduct. Target. Ther..

[46] Knopf P., Stowbur D., Hoffmann S.H.L., Hermann N., Maurer A., Bucher V., Poxleitner M., Tako B., Sonanini D., Krishnamachary B. (2023). Acidosis-Mediated Increase in IFN-γ-Induced PD-L1 Expression on Cancer Cells as an Immune Escape Mechanism in Solid Tumors. Mol. Cancer.

[47] Pustylnikov S., Costabile F., Beghi S., Facciabene A. (2018). Targeting Mitochondria in Cancer: Current Concepts and Immunotherapy Approaches. Transl. Res..

[48] Huang L., Xu H., Peng G. (2018). TLR-Mediated Metabolic Reprogramming in the Tumor Microenvironment: Potential Novel Strategies for Cancer Immunotherapy. Cell. Mol. Immunol..

[49] Zhao H., Raines L.N., Huang S.C.-C. (2020). Carbohydrate and Amino Acid Metabolism as Hallmarks for Innate Immune Cell Activation and Function. Cells.

[50] Gong D., Chen M., Wang Y., Shi J., Hou Y. (2022). Role of Ferroptosis on Tumor Progression and Immunotherapy. Cell Death Discov..

[51] Wang W., Green M., Choi J.E., Gijón M., Kennedy P.D., Johnson J.K., Liao P., Lang X., Kryczek I., Sell A. (2019). CD8^+^ T Cells Regulate Tumour Ferroptosis during Cancer Immunotherapy. Nature.

[52] Dai Z., Liu J., Zeng L., Shi K., Peng X., Jin Z., Zheng R., Zeng C. (2025). Targeting Ferroptosis in Cancer Therapy: Mechanisms, Strategies, and Clinical Applications. Cell Investig..

[53] Gao M., Yu W., Xi Z., Zhang Z., Fan X., Wang X. (2025). Recent Update on the Discovery of Indoleamine-2,3-Dioxygenase 1 Inhibitors Targeting Cancer Immunotherapy. Eur. J. Med. Chem..

[54] Jin S., Plikus M.V., Nie Q. (2025). CellChat for Systematic Analysis of Cell–Cell Communication from Single-Cell Transcriptomics. Nat. Protoc..

[55] Liang Y., Zhang R., Zhang L., Xie P. (2023). DrugChat: Towards Enabling ChatGPT-like Capabilities on Drug Molecule Graphs. arXiv.

[56] Liu T., Chu T., Luo X., Zhao H. (2025). Building a Unified Model for Drug Synergy Analysis Powered by Large Language Models. Nat. Commun..

[57] Sang S., Sun Z., Zheng W., Wang W., Islam M.T., Chen Y., Yuan Q., Cheng C., Xi S., Han Z. (2025). TME-Guided Deep Learning Predicts Chemotherapy and Immunotherapy Response in Gastric Cancer with Attention-Enhanced Residual Swin Transformer. Cell Rep. Med..

[58] Li X., Pan B., He Y., Wang Z., Tang Y., Zhang Y., Wang L., Han J. (2025). PathHDNN: A Pathway Hierarchical-Informed Deep Neural Network Framework for Predicting Immunotherapy Response and Mechanism Interpretation. Genome Med..

[59] Min J.H., Chen P.-J., Qureshi T.A., Javed S., Xie Y., Azab L., Wang L., Kim H., Li D., Yang J.D. (2025). Prediction of Immunotherapy Response in Hepatocellular Carcinoma Patients Using Pretreatment CT Images. Diagnostics.

