# Supplementary material for: ICIsc: A Deep Learning Framework for Predicting Immune Checkpoint Inhibitor Response by Integrating scRNA-Seq and Protein Language Models
Source: Bioengineering (Basel). 2026 Feb 6;13(2):187. doi: 10.3390/bioengineering13020187 (PMC12937945; doi:10.3390/bioengineering13020187)
Supplement: Supplementary file 1 [file bioengineering-13-00187-s001.zip › bioengineering-4120741-supplementary.pdf]

ICIsc: A deep learning framework for predicting immune checkpoint inhibitor response by integrating scRNA-seq and protein language models

Zhenyu Jin[1 †], Di Zhang[1 †], Luonan Chen[*2,1]

1. Key Laboratory of Systems Health Science of Zhejiang Province, School of Life Science, Hangzhou Institute for Advanced Study, University of Chinese Academy of Sciences, Chinese Academy of Sciences, Hangzhou, 310024, China
2. School of Mathematical Sciences and School of AI, Shanghai Jiao Tong University, Shanghai, 200240, China
† Co-first Author.
* Corresponding Author:
Luonan Chen, lnchen@sjtu.edu.cn

Keywords: Deep learning; Attention network; Checkpoint inhibitors; Immunotherapy response; Single-cell RNA sequencing; Single simple network

Supplementary Figure S1. Kaplan-Meier survival analysis of the 6 genes in the signature using bulk RNA-seq data across various cancer types. The survival curves illustrate the association between high and low expression of each gene. Plots include survival for genes – *CXCL13*, *IFITM2*, *STAT1*, *CXCL10*, *HLA-B*, *CD38*.

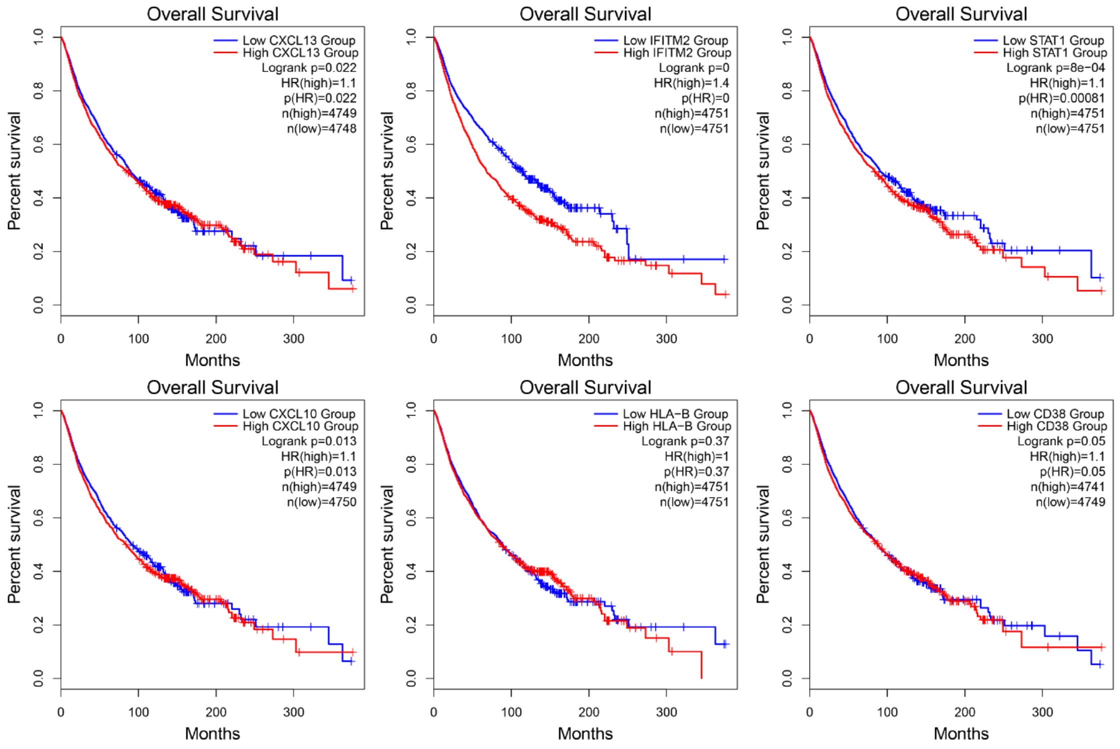

Supplementary Figure S2. Violin plot showing ferroptosis-related gene set scores in responders and non-responders. P-value was calculated by Wilcoxon test; * P < 0.05.

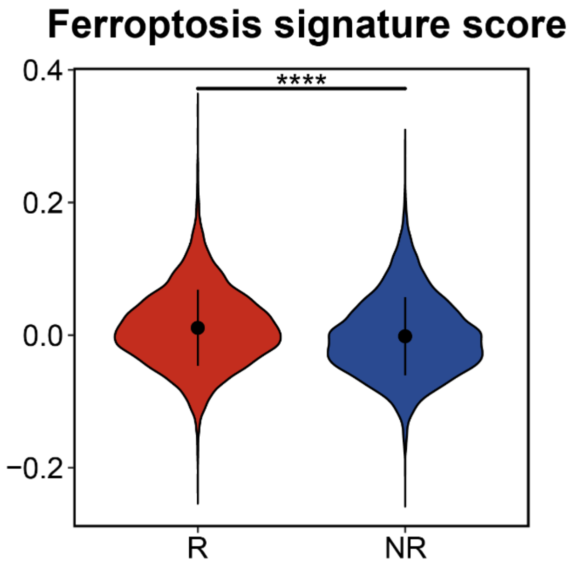

Supplementary Figure S3. Violin plot showing amino acid metabolism–related gene set scores in responders and non-responders. P-value was calculated by Wilcoxon test; * P < 0.05.

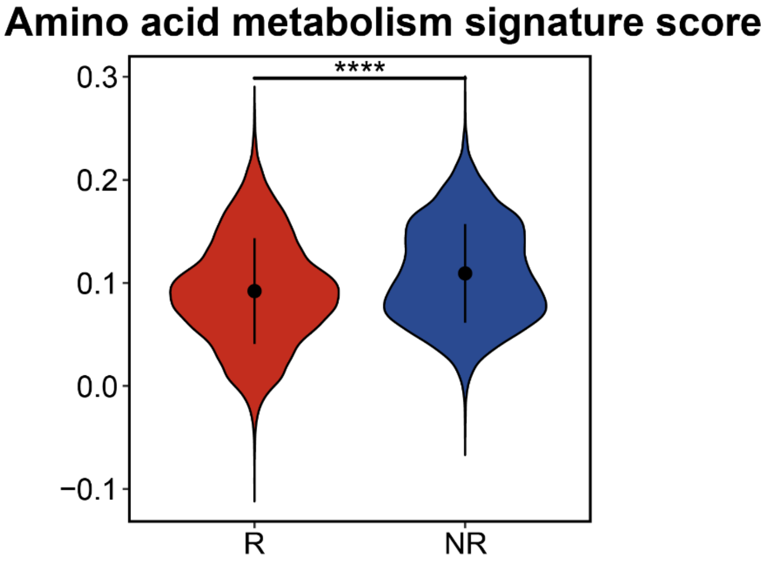

Supplementary Figure S4. Boxplots showing the expression levels of selected feature genes from the ferroptosis and amino acid metabolism pathways in responders and non-responders. P-value was calculated by Wilcoxon test; * P < 0.05.

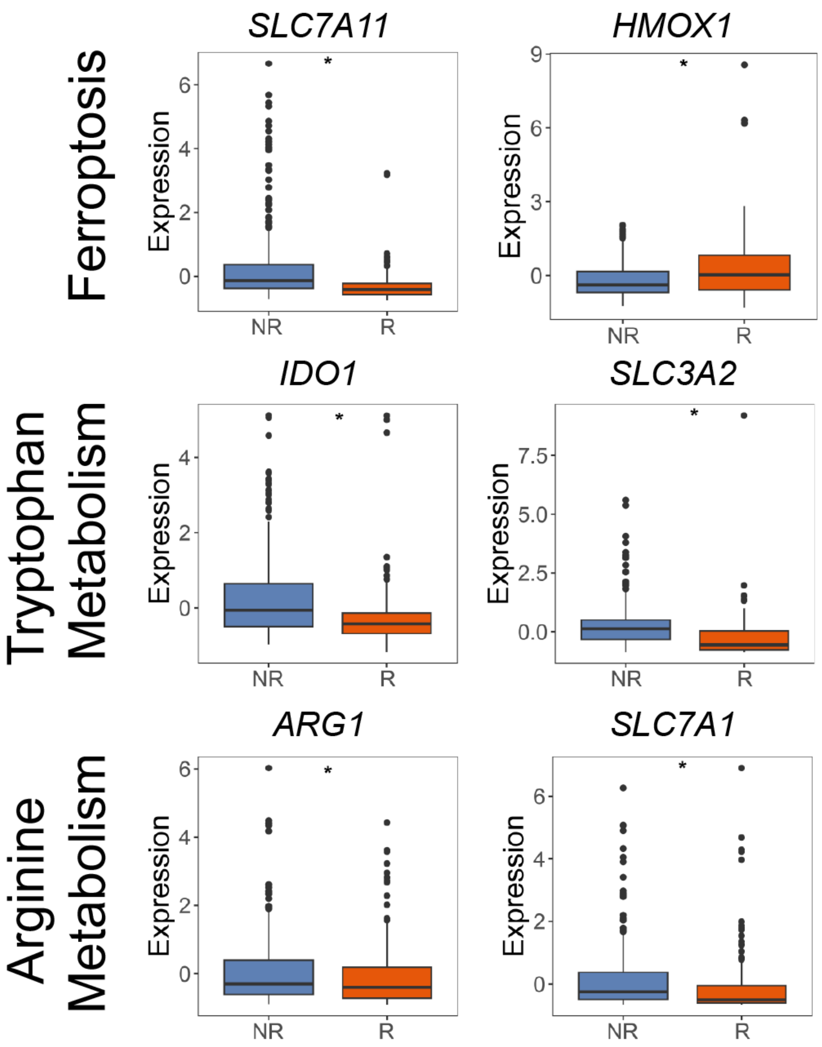

Supplementary Figure S5. Dot plot showing the expression of cell type specific marker. genes in different cell types.

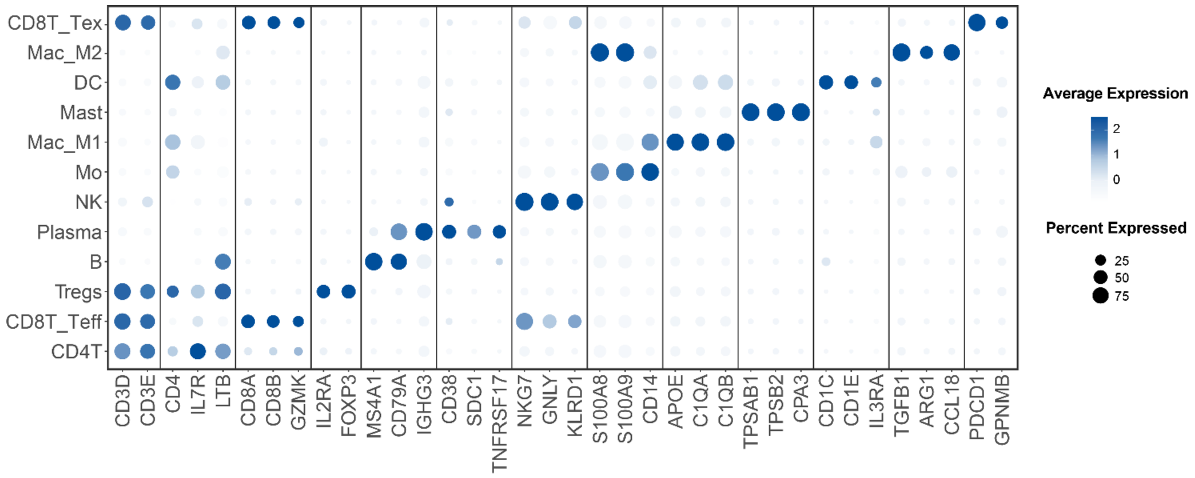

Supplementary Figure S6. Fraction of immune cells originating from each patient in GSE123814.

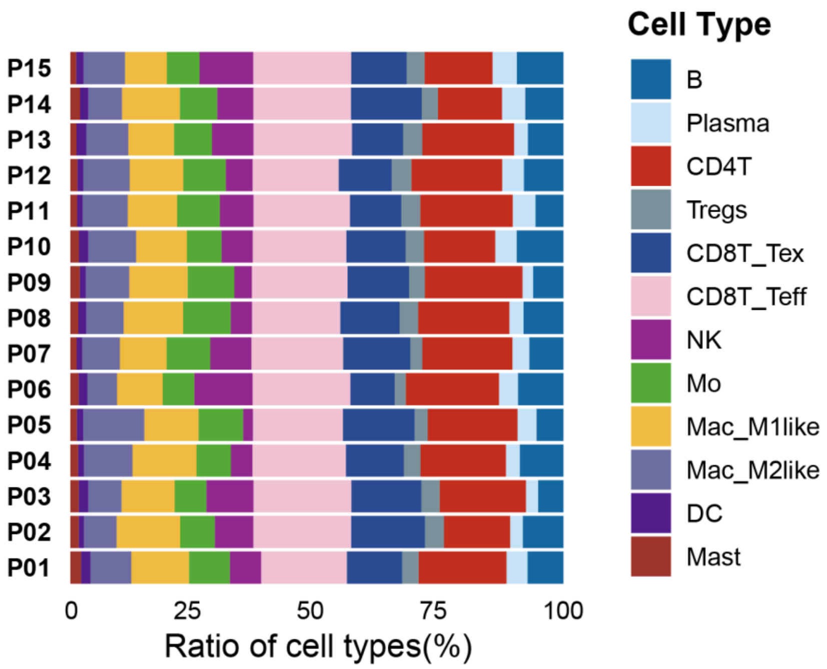

Supplementary Figure S7. Box plot showing the differences in the interactions strength of between Mac_M2like and other cell types in responders and non-responders. P-value was calculated by Wilcoxon test; * P < 0.05.

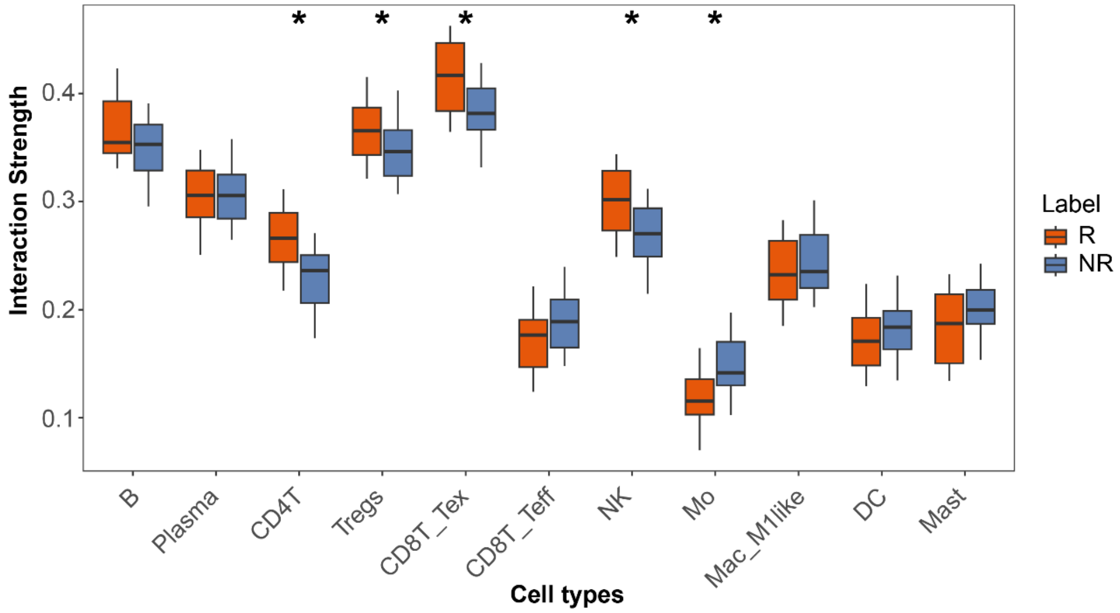

Supplementary Table S1. Summary of datasets.

		Dataset ID	Cancer Type	PMID	Therapy	Dataset Size	Responder Number	Non-Responder Number
Bluk	Train Sets	GBM-PRJNA482620	Glioblastoma	30996326	anti-PD-1	34	17	17
		HNSC-GSE93157	Squamous head and neck	28487385	anti-PD-1	5	2	3
		LUSC-GSE93157	Squamous-lung-carcinoma	28487385	anti-PD-1	13	3	10
		Melanoma-GSE145996	Melanoma	32708981	anti-PD1	4	2	2
		Melanoma-GSE115821	Melanoma	30127394	anti-CTLA-4, anti-PD-1, and anti-CTLA-4+anti-PD-1	37	3	34
		Melanoma-GSE78220	Melanoma	28129544	anti-PD-1	28	15	13
		Melanoma-GSE91061	Melanoma	29033130	anti-PD-1	109	20	78
		Melanoma-GSE93157	Melanoma	28487385	anti-PD-1	25	9	16
		Melanoma-Nathanson_2017	Melanoma	27956380	anti-CTLA-4	24	8	16
		Melanoma-phs000452	Melanoma	26359337	anti-PD-1	153	63	90
		NSCLC-GSE126044	Non small-cell lung cancer	32879421	anti-PD-1	7	2	5

		NSCLC-GSE135222	Non small-cell lung cancer	31537801	anti-PD-1	27	8	19
		RCC-Braun_2020	Renal cell carcinoma	32472114	anti-PD-1 and EVEROLIMUS	311	44	237
		RCC-GSE67501	Renal cell carcinoma	27491898	anti-PD-1	11	4	7
		STAD-PRJEB25780	Stomach adenocarcinoma	30013197	anti-PD-1	78	21	57
	Test Sets	Melanoma-PRJEB23709	Melanoma	30753825	anti-PD-1 and anti-CTLA-4+anti-PD-1	91	49	42
		nonsqNSCLC-GSE93157	Non small-cell lung cancer	28487385	anti-PD-1	22	6	16
Single Cell	Train Sets	BCC-GSE123814	Basal cell carcinoma	31359002	anti-PD-1	15	8	7
		TNBC-GSE169246	Triple-negative breast cancer	34653365	anti-PD-1	11	4	7
		Melanoma-phs001680	Melanoma	30388456	anti-CTLA-4, anti-PD-1, and anti-CTLA-4+anti-PD-1	48	17	31
		NSCLC-GSE173351	Non small-cell lung cancer	34290408	anti-PD-1	57	21	36
	Test Sets	BC-EGAS00001004809	Breast cancer	33958794	anti-PD-1	80	33	47

Supplementary Table S2. Immune checkpoint inhibitors drug protein sequences.

Drug Name	Heavy Chain Sequence	Light Chain Sequence
Nivolumab	QVQLVESGGGVVQPGRSLRLDCKASGITFSNSGMHWVR<br>QAPGKGLEWVAVIWYDGSKRYYADSVKGRFTISRDNSK<br>NTLFLQMNSLRAEDTAVYYCATNDDYWGQGTLVTVSSA<br>STKGPSVFPLAPCSRSTSESTAALGCLVKDYFPEPVTVSW<br>NSGALTSGVHTFPAVLQSSGLYSLSSVVTVPSSSLGTKTY<br>TCNVDHKPSNTKVDKRVESKYGPPCPPCPAPEFLGGPSVF<br>LFPPKPKDTLMISRTPEVTCVVVDVSQEDPEVQFNWYVD<br>GVEVHNAKTKPREEQFNSTYRVVSVLTVLHQDWLNGKE<br>YKCKVSNKGLPSSIEKTISKAKGQPREPQVYTLPPSQEEM<br>TKNQVSLTCLVKGFYPSDIAVEWESNGQPENNYKTTPPV<br>LDSDGSFFLYSRLTVDKSRWQEGNVFSCSVMHEALHNH<br>YTQKSLSLSLGK	EIVLTQSPATLSLSPGERATLSCRASQSVSSYLAWYQQKPG<br>QAPRLLIYDASNRATGIPARFSGSGSGTDFTLTISSLEPEDFA<br>VYYCQQSSNWPRTFGQGTKVEIKRTVAAPSVFIFPPSDEQL<br>KSGTASVVCLLNNFYPREAKVQWKVDNALQSGNSQESVTE<br>QDSKDSTYSLSSTLTLSKADYEKHKVYACEVTHQGLSSPVT<br>KSFNRGEC
Pembrolizumab	QVQLVQSGVEVKKPGASVKVSCKASGYTFTNYYMYWV<br>RQAPGQGLEWMGGINPSNGGTNFNEKFKNRVTLTTDSST<br>TTAYMELKSLQFDDTAVYYCARRDYRFDMGFDYWGQG<br>TTVTVSSASTKGPSVFPLAPCSRSTSESTAALGCLVKDYFP<br>EPVTVSWNSGALTSGVHTFPAVLQSSGLYSLSSVVTVPSS<br>SLGTKTYTCNVDHKPSNTKVDKRVESKYGPPCPPCPAPEF<br>LGGPSVFLFPPKPKDTLMISRTPEVTCVVVDVSQEDPEVQ<br>FNWYVDGVEVHNAKTKPREEQFNSTYRVVSVLTVLHQD<br>WLNGKEYKCKVSNKGLPSSIEKTISKAKGQPREPQVYTLP<br>PSQEEMTKNQVSLTCLVKGFYPSDIAVEWESNGQPENNY	EIVLTQSPATLSLSPGERATLSCRASKGVSTSGYSYLHWYQ<br>QKPGQAPRLLIYLASYLESGVPARFSGSGSGTDFTLTISSLEP<br>EDFAVYYCQHSRDLPLTFGGGTKVEIKRTVAAPSVFIFPPSD<br>EQLKSGTASVVCLLNNFYPREAKVQWKVDNALQSGNSQES<br>VTEQDSKDSTYSLSSTLTLSKADYEKHKVYACEVTHQGLSS<br>PVTKSFNRGEC

	KTTPPVLDSDGSFFLYSRLTVDKSRWQEGNVFSCSVMHE<br>ALHNHYTQKSLSLSLGK	
Ipilimumab	QVQLVESGGGVVQPGRSLRLSCAASGFTFSSYTMHWVR<br>QAPGKGLEWVTFISYDGNNKYYADSVKGRFTISRDNSKN<br>TLYLQMNSLRAEDTAIYYCARTGWLGPFDYWGQGTLVT<br>VSSASTKGPSVFPLAPSSKSTSGGTAALGCLVKDYFPEPV<br>TVSWNSGALTSGVHTFPAVLQSSGLYSLSSVVTVPSSSLG<br>TQTYICNVNHKPSNTKVDKRVEPKSCDKTHTCPPCPAPEL<br>LGGPSVFLFPPKPKDTLMISRTPEVTCVVVDVSHEDPEVK<br>FNWYVDGVEVHNAKTKPREEQYNSTYRVVSVLTVLHQD<br>WLNGKEYKCKVSNKALPAPIEKTISKAKGQPREPQVYTL<br>PPSRDELTKNQVSLTCLVKGFYPSDIAVEWESNGQPENN<br>YKTTPPVLDSDGSFFLYSKLTVDKSRWQQGNVFSCSVMH<br>EALHNHYTQKSLSLSPGK	EIVLTQSPGTLSLSPGERATLSCRASQSVGSSYLAWYQQKP<br>GQAPRLLIYGAFSRATGIPDRFSGSGSGTDFTLTISRLEPEDF<br>AVYYCQQYGSSPWTFGQGTKVEIKRTVAAPSVFIFPPSDEQ<br>LKSGTASVVCLLNNFYPREAKVQWKVDNALQSGNSQESVT<br>EQDSKDSTYSLSSTLTLSKADYEKHKVYACEVTHQGLSSPV<br>TKSFNRGEC
Cemiplimab	EVQLLESGGVLVQPGGSLRLSCAASGFTFSNFGMTWVRQ<br>APGKGLEWVSGISGGGRDTYFADSVKGRFTISRDNSKNT<br>LYLQMNSLKGEDTAVYYCVKWGNIYFDYWGQGTLVTV<br>SSASTKGPSVFPLAPCSRSTSESTAALGCLVKDYFPEPVTV<br>SWNSGALTSGVHTFPAVLQSSGLYSLSSVVTVPSSSLGTK<br>TYTCNVDHKPSNTKVDKRVESKYGPPCPPCPAPEFLGGPS<br>VFLFPPKPKDTLMISRTPEVTCVVVDVSQEDPEVQFNWY<br>VDGVEVHNAKTKPREEQFNSTYRVVSVLTVLHQDWLNG	DIQMTQSPSSLSASVGDSITITCRASLSINTFLNWYQQKPGK<br>APNLLIYAASSLHGGVPSRFSGSGSGTDFTLTIRTLQPEDFAT<br>YYCQQSSNTPFTFGPGTVVDFRRTVAAPSVFIFPPSDEQLKS<br>GTASVVCLLNNFYPREAKVQWKVDNALQSGNSQESVTEQ<br>DSKDSTYSLSSTLTLSKADYEKHKVYACEVTHQGLSSPVTK<br>SFNRGEC

	KEYKCKVSNKGLPSSIEKTISKAKGQPREPQVYTLPPSQEE<br>MTKNQVSLTCLVKGFYPSDIAVEWESNGQPENNYKTTPP<br>VLDSDGSFFLYSRLTVDKSRWQEGNVFSCSVMHEALHN<br>HYTQKSLSLSLGK	
Atezolizumab	EVQLVESGGGLVQPGGSLRLSCAASGFTFSDSWIHWVRQ<br>APGKGLEWVAWISPYGGSTYYADSVKGRFTISADTSKNT<br>AYLQMNSLRAEDTAVYYCARRHWPGGFDYWGQGTLVT<br>VSSASTKGPSVFPLAPSSKSTSGGTAALGCLVKDYFPEPV<br>TVSWNSGALTSGVHTFPAVLQSSGLYSLSSVVTVPSSSLG<br>TQTYICNVNHKPSNTKVDKKVEPKSCDKTHTCPPCPAPEL<br>LGGPSVFLFPPKPKDTLMISRTPEVTCVVVDVSHEDPEVK<br>FNWYVDGVEVHNAKTKPREEQYASTYRVVSVLTVLHQD<br>WLNGKEYKCKVSNKALPAPIEKTISKAKGQPREPQVYTL<br>PPSREEMTKNQVSLTCLVKGFYPSDIAVEWESNGQPENN<br>YKTTPPVLDSDGSFFLYSKLTVDKSRWQQGNVFSCSVMH<br>EALHNHYTQKSLSLSPGK	DIQMTQSPSSLSASVGDRVTITCRASQDVSTAVAWYQQKPG<br>KAPKLLIYSASFLYSGVPSRFSGSGSGTDFTLTISSLQPEDFA<br>TYYCQQYLYHPATFGQGTKVEIKRTVAAPSVFIFPPSDEQL<br>KSGTASVVCLLNNFYPREAKVQWKVDNALQSGNSQESVTE<br>QDSKDSTYSLSSTLTLSKADYEKHKVYACEVTHQGLSSPVT<br>KSFNRGEC

Supplementary Table S3. Gene sets associated with the tumor microenvironment and immunotherapy.

Feature ID	OrginalName	OriginalPublications	Marker Classification
P_001	aDC	PMID: 29628290; 24138885	Tumor-infiltrated cells
P_002	aDC	PMID: 29141660	Tumor-infiltrated cells
P_003	Angiogenesis	PMID: 29628290; 27855702	Anti-tumor or pro-tumor gene sets
P_004	Angiogenesis	PMID: 34019806	Anti-tumor or pro-tumor gene sets
P_005	Antitumor CY	PMID: 34019806	Anti-tumor or pro-tumor gene sets
P_006	APM1	PMID: 29628290; 27855702	Anti-tumor or pro-tumor gene sets
P_007	APM2	PMID: 29628290; 27855702	Anti-tumor or pro-tumor gene sets
P_008	AZ	PMID: 28052254	ICB predictor or signature
P_009	B.cell.memory	PMID: 31061481	Tumor-infiltrated cells
P_010	B.cell.naive	PMID: 31061481	Tumor-infiltrated cells
P_011	B.cell.plasma	PMID: 31061481	Tumor-infiltrated cells
P_012	B.cell	PMID: 32124323; 32124324	Tumor-infiltrated cells
P_013	B.cell	PMID: 32124323; 31126321	Tumor-infiltrated cells
P_014	B.cell	PMID: 32124323; 32442275	Tumor-infiltrated cells
P_015	B cells	PMID: 29628290; 24138885	Tumor-infiltrated cells
P_016	B-cells	PMID: 29141660	Tumor-infiltrated cells
P_017	B lineage	PMID: 27765066	Tumor-infiltrated cells
P_018	Bcell	PMID: 32274301	Tumor-infiltrated cells
P_019	B_cell_PCA_16704732	PMID: 29628290; 24516633; 16704732	Anti-tumor or pro-tumor gene sets
P_020	B_cells	PMID: 31641033	Tumor-infiltrated cells
P_021	B cells	PMID: 34019806	Tumor-infiltrated cells
P_022	Basophils	PMID: 29141660	Tumor-infiltrated cells
P_023	Bcell_21978456	PMID: 29628290; 24516633; 21978456	Anti-tumor or pro-tumor gene sets

P_024	Bcell_mg_IGJ	PMID: 29628290; 24516633	Anti-tumor or pro-tumor gene sets
P_025	Bcell_receptors_score	PMID: 29628290; 24516633	Anti-tumor or pro-tumor gene sets
P_026	Buck14_score	PMID: 29628290; 24516633	Anti-tumor or pro-tumor gene sets
P_027	Cancer-associated fibroblasts	PMID: 34019806	Tumor-infiltrated cells
P_028	CAF	PMID: 30127393	Tumor-infiltrated cells
P_029	Cancer.associated.fibroblast	PMID: 32124323; 32124324	Tumor-infiltrated cells
P_030	CD103neg_mean_25446897	PMID: 29628290; 24516633; 25446897	Anti-tumor or pro-tumor gene sets
P_031	CD103pos_CD103neg_ratio_25446897	PMID: 29628290; 24516633; 25446897	Anti-tumor or pro-tumor gene sets
P_032	CD103pos_mean_25446897	PMID: 29628290; 24516633; 25446897	Anti-tumor or pro-tumor gene sets
P_033	CD274	PMID: 30127393	Anti-tumor or pro-tumor gene sets
P_034	CD4+ memory T-cells	PMID: 29141660	Tumor-infiltrated cells
P_035	CD4+ naive T-cells	PMID: 29141660	Tumor-infiltrated cells
P_036	CD4+ T-cells	PMID: 29141660	Tumor-infiltrated cells
P_037	CD4+ Tcm	PMID: 29141660	Tumor-infiltrated cells
P_038	CD4+ Tem	PMID: 29141660	Tumor-infiltrated cells
P_039	CD4_naive	PMID: 32274301	Tumor-infiltrated cells
P_040	CD4_T	PMID: 32274301	Tumor-infiltrated cells
P_041	CD68	PMID: 29628290; 24516633	Anti-tumor or pro-tumor gene sets
P_042	CD8+ naive T-cells	PMID: 29141660	Tumor-infiltrated cells
P_043	CD8+ T-cells	PMID: 29141660	Tumor-infiltrated cells
P_044	CD8+ Tcm	PMID: 29141660	Tumor-infiltrated cells
P_045	CD8+ Tem	PMID: 29141660	Tumor-infiltrated cells
P_046	CD8 T cells	PMID: 27765066	Tumor-infiltrated cells
P_047	CD8 T cells	PMID: 29628290; 24138885	Tumor-infiltrated cells
P_048	CD8_CD68_ratio	PMID: 29628290; 24516633	Anti-tumor or pro-tumor gene sets
P_049	CD8_naive	PMID: 32274301	Tumor-infiltrated cells

P_050	CD8_PCA_16704732	PMID: 29628290; 24516633; 16704732	Anti-tumor or pro-tumor gene sets
P_051	CD8_T	PMID: 32274301	Tumor-infiltrated cells
P_052	CD8	PMID: 30127393	Anti-tumor or pro-tumor gene sets
P_053	CD8A	PMID: 29628290; 24516633	Anti-tumor or pro-tumor gene sets
P_054	cDC	PMID: 29141660	Tumor-infiltrated cells
P_055	Central_memory	PMID: 32274301	Tumor-infiltrated cells
P_056	CHANG_CORE_SERUM_RESPONSE_UP	PMID: 29628290; 24516633	Anti-tumor or pro-tumor gene sets
P_057	Checkpoint molecules	PMID: 34019806	Anti-tumor or pro-tumor gene sets
P_058	CK12_score	PMID: 29628290; 24516633	Anti-tumor or pro-tumor gene sets
P_059	Class-switched memory B-cells	PMID: 29141660	Tumor-infiltrated cells
P_060	Co-activation molecules	PMID: 34019806	Anti-tumor or pro-tumor gene sets
P_061	CP	PMID: 28052254	Anti-tumor or pro-tumor gene sets
P_062	CSF1_response	PMID: 29628290; 24516633	Anti-tumor or pro-tumor gene sets
P_063	CSR_Activated_15701700	PMID: 29628290; 24516633; 15701700	Anti-tumor or pro-tumor gene sets
P_064	CTLA4_data	PMID: 29628290; 24516633	Anti-tumor or pro-tumor gene sets
P_065	Cytotoxic cells	PMID: 29628290; 24138885	Tumor-infiltrated cells
P_066	Cytotoxic lymphocytes	PMID: 27765066	Tumor-infiltrated cells
P_067	Cytotoxic_cells	PMID: 31641033	Tumor-infiltrated cells
P_068	Cytotoxic	PMID: 32274301	Tumor-infiltrated cells
P_069	DAP12_data	PMID: 29628290; 24516633	Anti-tumor or pro-tumor gene sets
P_070	DC	PMID: 32274301	Tumor-infiltrated cells
P_071	DC	PMID: 29628290; 24138885	Tumor-infiltrated cells
P_072	DC	PMID: 29141660	Tumor-infiltrated cells
P_073	Dendritic_cells	PMID: 31641033	Tumor-infiltrated cells
P_074	Dysfunction	PMID: 30127393	Anti-tumor or pro-tumor gene sets
P_075	EC	PMID: 28052254	Anti-tumor or pro-tumor gene sets

P_076	Effector cells	PMID: 34019806	Anti-tumor or pro-tumor gene sets
P_077	Effector_memory	PMID: 32274301	Tumor-infiltrated cells
P_078	EMT signature	PMID: 34019806	Anti-tumor or pro-tumor gene sets
P_079	Endothelial.cell	PMID: 32124323; 32124324	Tumor-infiltrated cells
P_080	Endothelial cells	PMID: 27765066	Tumor-infiltrated cells
P_081	Endothelial cells	PMID: 29141660	Tumor-infiltrated cells
P_082	Endothelial	PMID: 31641033	Tumor-infiltrated cells
P_083	Endothelium	PMID: 34019806	Tumor-infiltrated cells
P_084	Eosinophil	PMID: 31061481	Tumor-infiltrated cells
P_085	Eosinophils	PMID: 31641033	Tumor-infiltrated cells
P_086	Eosinophils	PMID: 29628290; 24138885	Tumor-infiltrated cells
P_087	Eosinophils	PMID: 29141660	Tumor-infiltrated cells
P_088	Epithelial cells	PMID: 29141660	Tumor-infiltrated cells
P_089	Exclusion	PMID: 30127393	Anti-tumor or pro-tumor gene sets
P_090	Exhausted	PMID: 32274301	Tumor-infiltrated cells
P_091	Fibroblasts	PMID: 31641033	Tumor-infiltrated cells
P_092	Fibroblasts	PMID: 27765066	Tumor-infiltrated cells
P_093	Fibroblasts	PMID: 29141660	Tumor-infiltrated cells
P_094	G_CD3E	PMID: 29628290; 23468608; 23596202	Anti-tumor or pro-tumor gene sets
P_095	G_CYTH4	PMID: 29628290; 23468608; 23596202	Anti-tumor or pro-tumor gene sets
P_096	G_GIMAP4	PMID: 29628290; 23468608; 23596202	Anti-tumor or pro-tumor gene sets
P_097	G_HLA-DPA1	PMID: 29628290; 23468608; 23596202	Anti-tumor or pro-tumor gene sets
P_098	G_LILRB4	PMID: 29628290; 23468608; 23596202	Anti-tumor or pro-tumor gene sets
P_099	G_SIGLEC9	PMID: 29628290; 23468608; 23596202	Anti-tumor or pro-tumor gene sets
P_100	G_SLAMF6	PMID: 29628290; 23468608; 23596202	Anti-tumor or pro-tumor gene sets
P_101	Gamma_delta	PMID: 32274301	Tumor-infiltrated cells

P_102	GP11_Immune_IFN	PMID: 29628290; 24516633; 21214954	Anti-tumor or pro-tumor gene sets
P_103	NA	PMID: 29628290; 24516633	Anti-tumor or pro-tumor gene sets
P_104	GRANS_PCA_16704732	PMID: 29628290; 24516633; 16704732	Anti-tumor or pro-tumor gene sets
P_105	Granulocyte traffic	PMID: 34019806	Anti-tumor or pro-tumor gene sets
P_106	HER2_Immune_PCA_18006808	PMID: 29628290; 24516633; 18006808	Anti-tumor or pro-tumor gene sets
P_107	ICR_ACT_SCORE	PMID: 29628290; 26967649; 23890060; 28344865	Anti-tumor or pro-tumor gene sets
P_108	ICR_INHIB_SCORE	PMID: 29628290; 26967649; 23890060; 28344865	Anti-tumor or pro-tumor gene sets
P_109	ICR_SCORE	PMID: 29628290; 26967649; 23890060; 28344865	Anti-tumor or pro-tumor gene sets
P_110	ICS5_score	PMID: 29628290; 24516633	Anti-tumor or pro-tumor gene sets
P_111	iDC	PMID: 29628290; 24138885	Tumor-infiltrated cells
P_112	iDC	PMID: 29141660	Tumor-infiltrated cells
P_113	IFIT3	PMID: 29628290; 23468608; 23596202	Anti-tumor or pro-tumor gene sets
P_114	IFN_21978456	PMID: 29628290; 24516633; 21978456	Anti-tumor or pro-tumor gene sets
P_115	IFNG_score_21050467	PMID: 29628290; 24516633; 21050467	Anti-tumor or pro-tumor gene sets
P_116	IFNG	PMID: 30127393	Anti-tumor or pro-tumor gene sets
P_117	IgG_19272155	PMID: 29628290; 24516633; 19272155	Anti-tumor or pro-tumor gene sets
P_118	IGG_Cluster_21214954	PMID: 29628290; 24516633; 21214954	Anti-tumor or pro-tumor gene sets
P_119	IL12_score_21050467	PMID: 29628290; 24516633; 21050467	Anti-tumor or pro-tumor gene sets
P_120	IL13_score_21050467	PMID: 29628290; 24516633; 21050467	Anti-tumor or pro-tumor gene sets
P_121	IL2_score_21050467	PMID: 29628290; 24516633; 21050467	Anti-tumor or pro-tumor gene sets
P_122	IL4_score_21050467	PMID: 29628290; 24516633; 21050467	Anti-tumor or pro-tumor gene sets
P_123	IL8_21978456	PMID: 29628290; 24516633; 21978456	Anti-tumor or pro-tumor gene sets
P_124	Immune_cell_Cluster_21214954	PMID: 29628290; 24516633; 21214954	Anti-tumor or pro-tumor gene sets

P_125	Immune_NSCLC_score	PMID: 29628290; 24516633	Anti-tumor or pro-tumor gene sets
P_126	Immune_Score	PMID: 31641033	Tumor-infiltrated cells
P_127	ImmuneScore	PMID: 29141660	Tumor-infiltrated cells
P_128	InfiltrationScore	PMID: 32274301	Tumor-infiltrated cells
P_129	Interferon_19272155	PMID: 29628290; 24516633; 19272155	Anti-tumor or pro-tumor gene sets
P_130	Interferon_Cluster_21214954	PMID: 29628290; 24516633; 21214954	Anti-tumor or pro-tumor gene sets
P_131	IPS	PMID: 28052254	ICB predictor or signature
P_132	IR7_score	PMID: 29628290; 24516633	Anti-tumor or pro-tumor gene sets
P_133	iTreg	PMID: 32274301	Tumor-infiltrated cells
P_134	Keratinocytes	PMID: 29141660	Tumor-infiltrated cells
P_135	LCK_19272155	PMID: 29628290; 24516633; 19272155	Anti-tumor or pro-tumor gene sets
P_136	LIexpression_score	PMID: 29628290; 24516633	Anti-tumor or pro-tumor gene sets
P_137	ly Endothelial cells	PMID: 29141660	Tumor-infiltrated cells
P_138	LYM	PMID: 29628290; 23468608; 23596202	Anti-tumor or pro-tumor gene sets
P_139	Lymph vessels	PMID: 29628290; 24138885	Tumor-infiltrated cells
P_140	LYMPHS_PCA_16704732	PMID: 29628290; 24516633; 16704732	Anti-tumor or pro-tumor gene sets
P_141	M1 signature	PMID: 34019806	Tumor-infiltrated cells
P_142	M2	PMID: 30127393	Tumor-infiltrated cells
P_143	Macrophage.M0	PMID: 31061481	Tumor-infiltrated cells
P_144	Macrophage.M1	PMID: 31061481	Tumor-infiltrated cells
P_145	Macrophage.M1	PMID: 32124323; 31126321	Tumor-infiltrated cells
P_146	Macrophage.M2	PMID: 31061481	Tumor-infiltrated cells
P_147	Macrophage.M2	PMID: 32124323; 31126321	Tumor-infiltrated cells
P_148	Macrophages	PMID: 31641033	Tumor-infiltrated cells
P_149	Macrophage and DC traffic	PMID: 34019806	Anti-tumor or pro-tumor gene sets
P_150	Macrophage	PMID: 32124323; 32124324	Tumor-infiltrated cells

P_151	Macrophage	PMID: 32274301	Tumor-infiltrated cells
P_152	Macrophages_M1	PMID: 31641033	Tumor-infiltrated cells
P_153	Macrophages_M2	PMID: 31641033	Tumor-infiltrated cells
P_154	Macrophages	PMID: 29628290; 24138885	Tumor-infiltrated cells
P_155	Macrophage	PMID: 32124323; 32442275	Tumor-infiltrated cells
P_156	Macrophages M1	PMID: 29141660	Tumor-infiltrated cells
P_157	Macrophages M2	PMID: 29141660	Tumor-infiltrated cells
P_158	Tumor-associated Macrophages	PMID: 34019806	Tumor-infiltrated cells
P_159	Macrophages	PMID: 29141660	Tumor-infiltrated cells
P_160	MAIT	PMID: 32274301	Tumor-infiltrated cells
P_161	Mast.cell.activated	PMID: 31061481	Tumor-infiltrated cells
P_162	Mast.cell.resting	PMID: 31061481	Tumor-infiltrated cells
P_163	Mast cells	PMID: 29628290; 24138885	Tumor-infiltrated cells
P_164	Mast cells	PMID: 29141660	Tumor-infiltrated cells
P_165	Mast_cells	PMID: 31641033	Tumor-infiltrated cells
P_166	Matrix remodeling	PMID: 34019806	Anti-tumor or pro-tumor gene sets
P_167	Matrix	PMID: 34019806	Anti-tumor or pro-tumor gene sets
P_168	MCD3_CD8_21214954	PMID: 29628290; 24516633; 21214954	Anti-tumor or pro-tumor gene sets
P_169	MDACC.FNA.1_20805453	PMID: 29628290; 24516633	Anti-tumor or pro-tumor gene sets
P_170	Immune Suppression by Myeloid Cells	PMID: 34019806	Tumor-infiltrated cells
P_171	MDSC	PMID: 30127393	Tumor-infiltrated cells
P_172	Myeloid cells traffic	PMID: 34019806	Anti-tumor or pro-tumor gene sets
P_173	Memory B-cells	PMID: 29141660	Tumor-infiltrated cells
P_174	Mesangial cells	PMID: 29141660	Tumor-infiltrated cells
P_175	MHC.I_19272155	PMID: 29628290; 24516633; 19272155	Anti-tumor or pro-tumor gene sets
P_176	MHC.II_19272155	PMID: 29628290; 24516633; 19272155	Anti-tumor or pro-tumor gene sets

P_177	MHC	PMID: 28052254	Anti-tumor or pro-tumor gene sets
P_178	MHC1_21978456	PMID: 29628290; 24516633; 21978456	Anti-tumor or pro-tumor gene sets
P_179	MHC2_21978456	PMID: 29628290; 24516633; 21978456	Anti-tumor or pro-tumor gene sets
P_180	MHCI	PMID: 34019806	Anti-tumor or pro-tumor gene sets
P_181	MHCII	PMID: 34019806	Anti-tumor or pro-tumor gene sets
P_182	MicroenvironmentScore	PMID: 29141660	Tumor-infiltrated cells
P_183	Minterferon_Cluster_21214954	PMID: 29628290; 24516633; 21214954	Anti-tumor or pro-tumor gene sets
P_184	Module11_Prolif_score	PMID: 29628290; 24516633	Anti-tumor or pro-tumor gene sets
P_185	Module3_IFN_score	PMID: 29628290; 24516633	Anti-tumor or pro-tumor gene sets
P_186	Module4_TcellBcell_score	PMID: 29628290; 24516633	Anti-tumor or pro-tumor gene sets
P_187	Module5_TcellBcell_score	PMID: 29628290; 24516633	Anti-tumor or pro-tumor gene sets
P_188	Monocyte	PMID: 31061481	Tumor-infiltrated cells
P_189	Monocyte	PMID: 32274301	Tumor-infiltrated cells
P_190	Monocyte	PMID: 32124323; 31126321	Tumor-infiltrated cells
P_191	Monocytes	PMID: 31641033	Tumor-infiltrated cells
P_192	Monocytes	PMID: 29141660	Tumor-infiltrated cells
P_193	Monocytic lineage	PMID: 27765066	Tumor-infiltrated cells
P_194	mv Endothelial cells	PMID: 29141660	Tumor-infiltrated cells
P_195	Myeloid.dendritic.cell.activated	PMID: 31061481	Tumor-infiltrated cells
P_196	Myeloid.dendritic.cell.resting	PMID: 31061481	Tumor-infiltrated cells
P_197	Myeloid.dendritic.cell	PMID: 32124323; 31126321	Tumor-infiltrated cells
P_198	Myeloid.dendritic.cell	PMID: 32124323; 32442275	Tumor-infiltrated cells
P_199	Myeloid dendritic cells	PMID: 27765066	Tumor-infiltrated cells
P_200	naive B-cells	PMID: 29141660	Tumor-infiltrated cells
P_201	Neutrophil	PMID: 31061481	Tumor-infiltrated cells
P_202	Neutrophils	PMID: 31641033	Tumor-infiltrated cells

P_203	Neutrophil	PMID: 32274301	Tumor-infiltrated cells
P_204	Neutrophils	PMID: 27765066	Tumor-infiltrated cells
P_205	Neutrophil	PMID: 32124323; 31126321	Tumor-infiltrated cells
P_206	Neutrophils	PMID: 29628290; 24138885	Tumor-infiltrated cells
P_207	Neutrophil signature	PMID: 34019806	Tumor-infiltrated cells
P_208	Neutrophil	PMID: 32124323; 32442275	Tumor-infiltrated cells
P_209	Neutrophils	PMID: 29141660	Tumor-infiltrated cells
P_210	NHI_5gene_score	PMID: 29628290; 24516633	Anti-tumor or pro-tumor gene sets
P_211	NK CD56bright cells	PMID: 29628290; 24138885	Tumor-infiltrated cells
P_212	NK CD56dim cells	PMID: 29628290; 24138885	Tumor-infiltrated cells
P_213	NK.cell.activated	PMID: 31061481	Tumor-infiltrated cells
P_214	NK.cell.resting	PMID: 31061481	Tumor-infiltrated cells
P_215	NK_cells	PMID: 31641033	Tumor-infiltrated cells
P_216	NK.cell	PMID: 32124323; 32124324	Tumor-infiltrated cells
P_217	NK.cell	PMID: 32124323; 31126321	Tumor-infiltrated cells
P_218	NK cells	PMID: 29628290; 24138885	Tumor-infiltrated cells
P_219	NK cells	PMID: 27765066	Tumor-infiltrated cells
P_220	NK cells	PMID: 29141660	Tumor-infiltrated cells
P_221	NK cells	PMID: 34019806	Tumor-infiltrated cells
P_222	NK	PMID: 32274301	Tumor-infiltrated cells
P_223	NKT	PMID: 32274301	Tumor-infiltrated cells
P_224	NKT	PMID: 29141660	Tumor-infiltrated cells
P_225	nTreg	PMID: 32274301	Tumor-infiltrated cells
P_226	Anti-PD-1 resistant melanoma	PMID: 30388455	ICB predictor or signature
P_227	Anti-PD-1 resistant melanoma .down	PMID: 30388455	ICB predictor or signature
P_228	Anti-PD-1 resistant melanoma .up	PMID: 30388455	ICB predictor or signature

P_229	AXL (Tirosh)	PMID: 30388455	Anti-tumor or pro-tumor gene sets
P_230	Ayers IFNg sig	PMID: 30388455	Anti-tumor or pro-tumor gene sets
P_231	Ayers immune sig	PMID: 30388455	Anti-tumor or pro-tumor gene sets
P_232	co-culture screen hits10	PMID: 30388455	Anti-tumor or pro-tumor gene sets
P_233	co-culture screen hits50	PMID: 30388455	Anti-tumor or pro-tumor gene sets
P_234	exc	PMID: 30388455	ICB predictor or signature
P_235	exc.down; exc.seed.down	PMID: 30388455	ICB predictor or signature
P_236	exc.up; exc.seed.up	PMID: 30388455	ICB predictor or signature
P_237	Fluidgm Panel A	PMID: 30388455	Anti-tumor or pro-tumor gene sets
P_238	Fluidgm Panel B	PMID: 30388455	Anti-tumor or pro-tumor gene sets
P_239	G1 S (Tirosh)	PMID: 30388455	Anti-tumor or pro-tumor gene sets
P_240	G2 M (Tirosh)	PMID: 30388455	Anti-tumor or pro-tumor gene sets
P_241	in-vivo screen GVAX vs TCRaKO depleted	PMID: 30388455	Anti-tumor or pro-tumor gene sets
P_242	in-vivo screen GVAX vs TCRaKO enriched	PMID: 30388455	Anti-tumor or pro-tumor gene sets
P_243	in-vivo screen GVAXPD1 vs TCRaKO depleted	PMID: 30388455	Anti-tumor or pro-tumor gene sets
P_244	in-vivo screen GVAXPD1 vs TCRaKO enriched	PMID: 30388455	Anti-tumor or pro-tumor gene sets
P_245	in-vivo screen TCRaKO vs invitro depleted	PMID: 30388455	Anti-tumor or pro-tumor gene sets
P_246	in-vivo screen TCRaKO vs invitro enriched	PMID: 30388455	Anti-tumor or pro-tumor gene sets
P_247	MAPKi resistant melanoma	PMID: 30388455	ICB predictor or signature
P_248	MAPKi resistant melanoma down	PMID: 30388455	ICB predictor or signature
P_249	MAPKi resistant melanoma up	PMID: 30388455	ICB predictor or signature
P_250	Melanoma cell cycle (Tirosh)	PMID: 30388455	Anti-tumor or pro-tumor gene sets
P_251	Melanoma cells (Tirosh)	PMID: 30388455	Anti-tumor or pro-tumor gene sets
P_252	MITF (Tirosh)	PMID: 30388455	Anti-tumor or pro-tumor gene sets

P_253	Nivolumab (molecular) resistant melanoma	PMID: 30388455	ICB predictor or signature
P_254	Nivolumab (molecular) resistant melanoma down	PMID: 30388455	ICB predictor or signature
P_255	Nivolumab (molecular) resistant melanoma up	PMID: 30388455	ICB predictor or signature
P_256	Nivolumab resistant melanoma	PMID: 30388455	ICB predictor or signature
P_257	Nivolumab resistant melanoma down	PMID: 30388455	ICB predictor or signature
P_258	Nivolumab resistant melanoma up	PMID: 30388455	ICB predictor or signature
P_259	On Nivolumab	PMID: 30388455	ICB predictor or signature
P_260	On Nivolumab down	PMID: 30388455	ICB predictor or signature
P_261	On Nivolumab up	PMID: 30388455	ICB predictor or signature
P_262	res	PMID: 30388455	ICB predictor or signature
P_263	res.down	PMID: 30388455	ICB predictor or signature
P_264	res.up	PMID: 30388455	ICB predictor or signature
P_265	resF	PMID: 30388455	ICB predictor or signature
P_266	resF.down	PMID: 30388455	ICB predictor or signature
P_267	resF minus T CD8	PMID: 30388455	ICB predictor or signature
P_268	resF.up	PMID: 30388455	ICB predictor or signature
P_269	Responders on Nivolumab	PMID: 30388455	ICB predictor or signature
P_270	Responders on Nivolumab down	PMID: 30388455	ICB predictor or signature
P_271	Responders on Nivolumab up	PMID: 30388455	ICB predictor or signature
P_272	resu	PMID: 30388455	ICB predictor or signature
P_273	resu.down	PMID: 30388455	ICB predictor or signature
P_274	resu.up	PMID: 30388455	ICB predictor or signature
P_275	TME B cell	PMID: 30388455	Tumor-infiltrated cells
P_276	TME CAF	PMID: 30388455	Tumor-infiltrated cells

P_277	TME Endo	PMID: 30388455	Tumor-infiltrated cells
P_278	TME immune	PMID: 30388455	Tumor-infiltrated cells
P_279	TME Macrophage	PMID: 30388455	Tumor-infiltrated cells
P_280	TME Mal	PMID: 30388455	Tumor-infiltrated cells
P_281	TME NK	PMID: 30388455	Tumor-infiltrated cells
P_282	TME stroma	PMID: 30388455	Tumor-infiltrated cells
P_283	TME T CD4	PMID: 30388455	Tumor-infiltrated cells
P_284	TME T CD4 exhausted	PMID: 30388455	Tumor-infiltrated cells
P_285	TME T CD4 naive	PMID: 30388455	Tumor-infiltrated cells
P_286	TME T CD4 Treg	PMID: 30388455	Tumor-infiltrated cells
P_287	TME T CD8	PMID: 30388455	Tumor-infiltrated cells
P_288	TME T CD8 cytotoxic	PMID: 30388455	Tumor-infiltrated cells
P_289	TME T CD8 exhausted	PMID: 30388455	Tumor-infiltrated cells
P_290	TME T CD8 naive	PMID: 30388455	Tumor-infiltrated cells
P_291	TME T cell	PMID: 30388455	Tumor-infiltrated cells
P_292	trt	PMID: 30388455	ICB predictor or signature
P_293	trt.down	PMID: 30388455	ICB predictor or signature
P_294	trt.up	PMID: 30388455	ICB predictor or signature
P_295	PD1_data	PMID: 29628290; 24516633	Anti-tumor or pro-tumor gene sets
P_296	PD1_PDL1_score	PMID: 29628290; 24516633	Anti-tumor or pro-tumor gene sets
P_297	pDC	PMID: 29628290; 24138885	Tumor-infiltrated cells
P_298	pDC	PMID: 29141660	Tumor-infiltrated cells
P_299	PDCD1	PMID: 30127393	Anti-tumor or pro-tumor gene sets
P_300	PDL1_data	PMID: 29628290; 24516633	Anti-tumor or pro-tumor gene sets
P_301	Pericytes	PMID: 29141660	Tumor-infiltrated cells
P_302	Plasma cells	PMID: 29141660	Tumor-infiltrated cells

P_303	Plasma_cells	PMID: 31641033	Tumor-infiltrated cells
P_304	pro B-cells	PMID: 29141660	Tumor-infiltrated cells
P_305	Tumor proliferation rate	PMID: 34019806	Anti-tumor or pro-tumor gene sets
P_306	Protumor CY	PMID: 34019806	Anti-tumor or pro-tumor gene sets
P_307	Rotterdam_ERneg_PCA_15721472	PMID: 29628290; 24516633; 15721472	Anti-tumor or pro-tumor gene sets
P_308	SC	PMID: 28052254	Anti-tumor or pro-tumor gene sets
P_309	Sebocytes	PMID: 29141660	Tumor-infiltrated cells
P_310	STAT1_19272155	PMID: 29628290; 24516633; 19272155	Anti-tumor or pro-tumor gene sets
P_311	STAT1_score	PMID: 29628290; 24516633	Anti-tumor or pro-tumor gene sets
P_312	StromaScore	PMID: 29141660	Tumor-infiltrated cells
P_313	T.cell.CD4...non.regulatory.	PMID: 32124323; 31126321	Tumor-infiltrated cells
P_314	T.cell.CD4..memory.activated	PMID: 31061481	Tumor-infiltrated cells
P_315	T.cell.CD4..memory.resting	PMID: 31061481	Tumor-infiltrated cells
P_316	T.cell.CD4.	PMID: 32124323; 32124324	Tumor-infiltrated cells
P_317	T.cell.CD4.	PMID: 32124323; 32442275	Tumor-infiltrated cells
P_318	T.cell.CD4..naive	PMID: 31061481	Tumor-infiltrated cells
P_319	T.cell.CD8.	PMID: 31061481	Tumor-infiltrated cells
P_320	T.cell.CD8.	PMID: 32124323; 32124324	Tumor-infiltrated cells
P_321	T.cell.CD8.	PMID: 32124323; 31126321	Tumor-infiltrated cells
P_322	T.cell.CD8.	PMID: 32124323; 32442275	Tumor-infiltrated cells
P_323	T.cell.follicular.helper	PMID: 31061481	Tumor-infiltrated cells
P_324	T.cell.regulatory..Tregs.	PMID: 32124323; 31126321	Tumor-infiltrated cells
P_325	T.cell.gamma.delta	PMID: 31061481	Tumor-infiltrated cells
P_326	T.cell.regulatory..Tregs.	PMID: 31061481	Tumor-infiltrated cells
P_327	T cells	PMID: 27765066	Tumor-infiltrated cells
P_328	T cells	PMID: 29628290; 24138885	Tumor-infiltrated cells

P_329	T helper cells	PMID: 29628290; 24138885	Tumor-infiltrated cells
P_330	T_cell_PCA_16704732	PMID: 29628290; 24516633; 16704732	Anti-tumor or pro-tumor gene sets
P_331	Effector cell traffic	PMID: 34019806	Anti-tumor or pro-tumor gene sets
P_332	T_cells_CD4	PMID: 31641033	Tumor-infiltrated cells
P_333	T_cells_CD8	PMID: 31641033	Tumor-infiltrated cells
P_334	T_cells_gamma_delta	PMID: 31641033	Tumor-infiltrated cells
P_335	T cells	PMID: 34019806	Tumor-infiltrated cells
P_336	Treg and Th2 traffic	PMID: 34019806	Anti-tumor or pro-tumor gene sets
P_337	T_regulatory_cells	PMID: 31641033	Tumor-infiltrated cells
P_338	TAMsurr_score	PMID: 29628290; 24516633	Anti-tumor or pro-tumor gene sets
P_339	TAMsurr_TcClassII_ratio	PMID: 29628290; 24516633	Anti-tumor or pro-tumor gene sets
P_340	TcClassII_score	PMID: 29628290; 24516633	Anti-tumor or pro-tumor gene sets
P_341	Tcell_21978456	PMID: 29628290; 24516633; 21978456	Anti-tumor or pro-tumor gene sets
P_342	Tcell_receptors_score	PMID: 29628290; 24516633	Anti-tumor or pro-tumor gene sets
P_343	Tcm cells	PMID: 29628290; 24138885	Tumor-infiltrated cells
P_344	Tem cells	PMID: 29628290; 24138885	Tumor-infiltrated cells
P_345	Tfh cells	PMID: 29628290; 24138885	Tumor-infiltrated cells
P_346	Tfh	PMID: 32274301	Tumor-infiltrated cells
P_347	Tgd cells	PMID: 29628290; 24138885	Tumor-infiltrated cells
P_348	Tgd cells	PMID: 29141660	Tumor-infiltrated cells
P_349	TGFB_PCA_17349583	PMID: 29628290; 24516633; 17349583	Anti-tumor or pro-tumor gene sets
P_350	TGFB_score_21050467	PMID: 29628290; 24516633; 21050467	Anti-tumor or pro-tumor gene sets
P_351	Th1 cells	PMID: 29628290; 24138885	Tumor-infiltrated cells
P_352	Th1 cells	PMID: 29141660	Tumor-infiltrated cells
P_353	Th1	PMID: 32274301	Tumor-infiltrated cells
P_354	Th1 signature	PMID: 34019806	Tumor-infiltrated cells

P_355	Th17 cells	PMID: 29628290; 24138885	Tumor-infiltrated cells
P_356	Th17	PMID: 32274301	Tumor-infiltrated cells
P_357	Th2 cells	PMID: 29628290; 24138885	Tumor-infiltrated cells
P_358	Th2.cells	PMID: 29141660	Tumor-infiltrated cells
P_359	Th2	PMID: 32274301	Tumor-infiltrated cells
P_360	Th2 signature	PMID: 34019806	Tumor-infiltrated cells
P_361	TIDE	PMID: 30127393	ICB predictor or signature
P_362	TIGS	PMID: 31767055	ICB predictor or signature
P_363	TIP_signature	PMID: 33523948	ICB predictor or signature
P_364	Tr1	PMID: 32274301	Tumor-infiltrated cells
P_365	Treg cells	PMID: 29628290; 24138885	Tumor-infiltrated cells
P_366	Treg	PMID: 34019806	Tumor-infiltrated cells
P_367	Tregs	PMID: 29141660	Tumor-infiltrated cells
P_368	TREM1_data	PMID: 29628290; 24516633	Anti-tumor or pro-tumor gene sets
P_369	Troester_WoundSig_19887484	PMID: 29628290; 24516633; 19887484	Anti-tumor or pro-tumor gene sets
P_370	uncharacterized.cell	PMID: 32124323; 32124324	Tumor-infiltrated cells
P_371	uncharacterized.cell	PMID: 32124323; 31126321	Tumor-infiltrated cells

Supplementary Table S4. Comparative Performance of ICIsc and Baseline Models in Bulk Test Cohorts with 95% Confidence Intervals and Statistical Significance Testing.

Model	Accuracy	Accuracy(95%CI)	p-value	AUROC	AUROC(95%CI)	p-value
RF	0.6545	0.5877-0.6822	0.0096	0.6606	0.5976-0.7021	0.0172
SVM	0.6611	0.6023-0.7007	0.0103	0.6572	0.5934-0.6998	0.0183
XGBoost	0.7272	0.6832-0.7774	0.0135	0.7110	0.6746-0.7553	0.0251
EN	0.6824	0.6172-0.7336	0.0097	0.6823	0.6431-0.7392	0.0227
LightGBM	0.6742	0.6254-0.7199	0.0124	0.6767	0.6228-0.7185	0.0196
IRnet	0.8126	0.7886-0.8741	0.0381	0.7131	0.6801-0.7447	0.0386
TIDE	0.4555	0.4069-0.5134	0.0056	0.4419	0.3999-0.4890	0.0122
iMLGAM	0.7981	0.7448-0.8445	0.0299	0.7005	0.6752-0.7491	0.0317
ICIsc	0.8671	0.7879–0.8932	/	0.8621	0.7822-0.8733	/

Supplementary Table S5. Comparative Performance of ICIsc and Baseline Models in Bulk Validation Cohorts with 95% Confidence Intervals and Statistical Significance Testing.

Model	Accuracy	Accuracy(95%CI)	p-value	AUROC	AUROC(95%CI)	p-value
RF	0.5383	0.4821-0.5636	0.0137	0.5340	0.4923-0.5774	0.0216
SVM	0.5344	0.4983-0.5774	0.0152	0.5722	0.5361-0.6142	0.0247
XGBoost	0.5900	0.5499-0.6348	0.0183	0.6621	0.6370-0.7003	0.0299
EN	0.5671	0.5302-0.5924	0.0122	0.5757	0.5396-0.6217	0.0254
LightGBM	0.5888	0.5366-0.6271	0.0125	0.6119	0.5833-0.6475	0.0304
IRnet	0.6272	0.5845-0.6683	0.0297	0.6554	0.6212-0.6874	0.0397
TIDE	0.4810	0.4440-0.5291	0.0104	0.4811	0.4577-0.5663	0.0153
iMLGAM	0.6634	0.6252-0.6997	0.0311	0.6821	0.6399-0.7258	0.0402
ICIsc	0.7953	0.7411-0.8207	/	0.7862	0.7448-0.8190	/

Supplementary Table S6. Comparative Performance of ICIsc and Baseline Models in Single-Cell Test Cohorts with 95% Confidence Intervals and Statistical Significance Testing.

Model	Accuracy	Accuracy(95%CI)	p-value	AUROC	AUROC(95%CI)	p-value
RF	0.7062	0.6652-0.7364	0.0213	0.6963	0.6578-0.7413	0.0236
SVM	0.7175	0.6749-0.7533	0.0295	0.6884	0.6437-0.7365	0.0232
XGBoost	0.7561	0.7318–0.7898	0.0302	0.7432	0.7083-0.7958	0.0193
EN	0.7323	0.6926–0.7619	0.0294	0.7159	0.6797-0.7622	0.0254
LightGBM	0.6931	0.6577-0.7236	0.0288	0.6778	0.6178-0.7298	0.0197
IRnet	0.8219	0.7531–0.9101	0.0377	0.7882	0.7406-0.8140	0.0410
TIDE	0.6135	0.5705–0.6406	0.0124	0.5871	0.5395-0.6284	0.0081
iMLGAM	0.8137	0.7731–0.8389	0.0389	0.7655	0.7124-0.7968	0.0321
ICIsc	0.8645	0.8189–0.9195	/	0.8421	0.8077-0.8926	/

Supplementary Table S7. Comparative Performance of ICIsc and Baseline Models in Single-Cell Validation Cohorts with 95% Confidence Intervals and Statistical Significance Testing.

Model	Accuracy	Accuracy(95%CI)	p-value	AUROC	AUROC(95%CI)	p-value
RF	0.5713	0.5172-0.6311	0.0131	0.5622	0.4899-0.5923	0.0181
SVM	0.5634	0.5355-0.6217	0.0188	0.5528	0.5002-0.6121	0.0201
XGBoost	0.6642	0.6021-0.7083	0.0247	0.6379	0.5871-0.6667	0.0233
EN	0.5816	0.5346-0.6588	0.0198	0.5494	0.4899-0.5874	0.0218
LightGBM	0.6127	0.5564-0.6924	0.0203	0.5925	0.5433-0.6348	0.0194
IRnet	0.6729	0.6149-0.7523	0.0285	0.6491	0.6013-0.6944	0.0381
TIDE	0.5891	0.5137-0.6376	0.0012	0.5631	0.5115-0.6030	0.0104
iMLGAM	0.6961	0.6322-0.7628	0.0337	0.6884	0.6355-0.7132	0.0298
ICIsc	0.8153	0.7766–0.8461	/	0.7965	0.7654-0.8335	/

Supplementary Table S8. Ablation experiment results of variant models in which one feature is removed for ICIsc-bulk model.

Model	Accuracy	AUROC	F1-Scroe
w/o ICI	0.7921	0.7759	0.6363
w/o Pathway-score	0.7837	0.7626	0.6132

Supplementary Table S9. Ablation experiment results of variant models for ICIsc model.

Model	Accuracy	AUROC	F1-score
w/o ICI	0.7833	0.7698	0.6693
w/o Celltype-Proportion	0.7792	0.7596	0.6873
u CellChat	0.8021	0.7699	0.7003
u Cell-cell-Correlation	0.7846	0.7647	0.6888

Supplementary Table S10. Fraction of immune cells originating from each patient in scRNA-seq datasets.

DataSet ID	Patient	B	Plasma	CD4T	CD8T_Tex	CD8T_Teff	Tregs	NK	Mac_M1like	Mac_M2like	Mo	DC	Mast
31359002	P01	0.0731	0.0423	0.1786	0.1123	0.1737	0.0332	0.0638	0.1172	0.0831	0.0826	0.0189	0.0212
	P02	0.0827	0.0257	0.1341	0.1498	0.1985	0.0385	0.0781	0.1290	0.0658	0.0704	0.0108	0.0166
	P03	0.0515	0.0247	0.1749	0.1418	0.1987	0.0374	0.0955	0.1079	0.0674	0.0642	0.0195	0.0165
	P04	0.0888	0.0281	0.1732	0.1173	0.1897	0.0338	0.0439	0.1297	0.0984	0.0697	0.0119	0.0155
	P05	0.0549	0.0383	0.1820	0.1457	0.1826	0.0266	0.0200	0.1102	0.1240	0.0903	0.0128	0.0126
	P06	0.0924	0.0385	0.1891	0.0896	0.1984	0.0227	0.1183	0.0925	0.0604	0.0642	0.0177	0.0162
	P07	0.0694	0.0342	0.1828	0.1361	0.1861	0.0245	0.0835	0.0949	0.0765	0.0885	0.0110	0.0125
	P08	0.0811	0.0287	0.1848	0.1204	0.1791	0.0378	0.0436	0.1206	0.0761	0.0962	0.0159	0.0157
	P09	0.0617	0.0216	0.1979	0.1249	0.1941	0.0322	0.0354	0.1187	0.0879	0.0946	0.0122	0.0188
	P10	0.0952	0.0434	0.1446	0.1203	0.1900	0.0370	0.0633	0.1028	0.0971	0.0703	0.0189	0.0171
	P11	0.0570	0.0460	0.1875	0.1041	0.1957	0.0387	0.0684	0.1003	0.0917	0.0864	0.0108	0.0134
	P12	0.0805	0.0439	0.1841	0.1072	0.1748	0.0402	0.0546	0.1086	0.0942	0.0862	0.0117	0.0140
	P13	0.0722	0.0280	0.1862	0.1037	0.2000	0.0389	0.0838	0.0926	0.0853	0.0775	0.0199	0.0119
	P14	0.0776	0.0471	0.1300	0.1435	0.1980	0.0329	0.0735	0.1171	0.0687	0.0758	0.0163	0.0195
	P15	0.0949	0.0490	0.1380	0.1124	0.1988	0.0363	0.1090	0.0850	0.0840	0.0662	0.0150	0.0114
34653365	P01	0.0770	0.0416	0.1516	0.1474	0.1826	0.0379	0.0298	0.1198	0.0992	0.0822	0.0136	0.0173
	P02	0.0960	0.0464	0.1762	0.0837	0.1908	0.0373	0.0660	0.1232	0.0817	0.0713	0.0146	0.0128
	P03	0.0536	0.0230	0.1751	0.1391	0.1992	0.0403	0.0089	0.1284	0.0970	0.0976	0.0186	0.0192
	P04	0.0870	0.0492	0.1890	0.1184	0.1624	0.0351	0.0348	0.1205	0.0794	0.0989	0.0111	0.0142
	P05	0.0681	0.0416	0.1723	0.1319	0.1982	0.0355	0.0776	0.0910	0.0749	0.0787	0.0158	0.0144
	P06	0.0706	0.0373	0.1432	0.1420	0.1981	0.0381	0.0629	0.1026	0.0857	0.0870	0.0176	0.0149
	P07	0.0627	0.0238	0.1761	0.1480	0.1934	0.0275	0.0760	0.1222	0.0795	0.0607	0.0183	0.0118
	P08	0.0748	0.0446	0.1518	0.1393	0.1810	0.0380	0.0956	0.0853	0.0922	0.0700	0.0138	0.0136
	P09	0.0716	0.0484	0.1977	0.1458	0.1749	0.0015	0.0777	0.0922	0.0794	0.0814	0.0129	0.0195
	P10	0.0529	0.0225	0.1945	0.1445	0.1884	0.0302	0.0865	0.0845	0.0839	0.0842	0.0117	0.0162
	P11	0.0706	0.0261	0.1685	0.1396	0.1862	0.0389	0.0980	0.0904	0.0784	0.0737	0.0104	0.0192
30388456	P01	0.0622	0.0335	0.1848	0.1162	0.1971	0.0381	0.1129	0.0881	0.0702	0.0696	0.0153	0.0120
	P02	0.0775	0.0309	0.1834	0.1253	0.1796	0.0367	0.0847	0.0853	0.0867	0.0812	0.0106	0.0181
	P03	0.0926	0.0478	0.1609	0.1428	0.1504	0.0387	0.0447	0.0988	0.0996	0.0882	0.0195	0.0160
	P04	0.0955	0.0254	0.1866	0.0984	0.1910	0.0357	0.0775	0.0982	0.0728	0.0891	0.0179	0.0119

P05	0.0628	0.0226	0.1593	0.1493	0.1987	0.0389	0.0725	0.1186	0.0627	0.0892	0.0123	0.0131
P06	0.0582	0.0426	0.1956	0.1373	0.1850	0.0107	0.0637	0.1101	0.0859	0.0790	0.0147	0.0172
P07	0.0720	0.0265	0.1457	0.1477	0.1992	0.0379	0.0890	0.0895	0.0683	0.0966	0.0115	0.0161
P08	0.0998	0.0279	0.1542	0.1164	0.1939	0.0381	0.1145	0.0918	0.0709	0.0682	0.0120	0.0123
P09	0.0581	0.0255	0.1759	0.1314	0.1998	0.0386	0.0413	0.1102	0.0951	0.0939	0.0146	0.0156
P10	0.0512	0.0298	0.1794	0.1380	0.1951	0.0359	0.0945	0.1185	0.0730	0.0612	0.0104	0.0130
P11	0.0949	0.0333	0.1482	0.1433	0.1838	0.0264	0.0719	0.0977	0.0797	0.0989	0.0101	0.0118
P12	0.0697	0.0495	0.1494	0.1344	0.1881	0.0390	0.0635	0.1244	0.0742	0.0795	0.0108	0.0175
P13	0.0985	0.0369	0.1938	0.1021	0.1926	0.0114	0.0690	0.0959	0.0941	0.0768	0.0117	0.0172
P14	0.0856	0.0351	0.1867	0.0956	0.1919	0.0367	0.0375	0.1278	0.0877	0.0833	0.0134	0.0187
P15	0.0707	0.0480	0.1303	0.1451	0.1980	0.0378	0.0946	0.0914	0.0910	0.0694	0.0137	0.0100
P16	0.0761	0.0442	0.1473	0.1267	0.1975	0.0388	0.0847	0.1285	0.0613	0.0682	0.0125	0.0142
P17	0.0575	0.0212	0.1636	0.1484	0.2170	0.0384	0.0538	0.1014	0.0875	0.0821	0.0155	0.0136
P18	0.0624	0.0275	0.1836	0.1167	0.1998	0.0390	0.0441	0.1041	0.0918	0.0995	0.0183	0.0132
P19	0.0733	0.0268	0.1874	0.1366	0.1930	0.0281	0.0252	0.1267	0.0795	0.0901	0.0160	0.0173
P20	0.0959	0.0281	0.1786	0.1334	0.1672	0.0321	0.0335	0.1198	0.0863	0.0916	0.0191	0.0144
P21	0.0539	0.0346	0.1635	0.1414	0.1987	0.0379	0.0708	0.1133	0.0722	0.0842	0.0195	0.0100
P22	0.0855	0.0278	0.1519	0.1264	0.1997	0.0383	0.0826	0.1033	0.0676	0.0868	0.0134	0.0167
P23	0.0926	0.0415	0.1698	0.1459	0.1984	0.0246	0.0097	0.1228	0.0756	0.0984	0.0105	0.0102
P24	0.0652	0.0399	0.1778	0.1346	0.1837	0.0340	0.0549	0.1158	0.0832	0.0804	0.0109	0.0196
P25	0.0672	0.0407	0.1490	0.1450	0.1998	0.0289	0.0465	0.1124	0.0954	0.0823	0.0180	0.0148
P26	0.0943	0.0436	0.1486	0.1060	0.1983	0.0388	0.0567	0.1183	0.0686	0.0963	0.0200	0.0105
P27	0.0642	0.0415	0.1601	0.1283	0.1989	0.0360	0.0796	0.1097	0.0770	0.0752	0.0172	0.0123
P28	0.0986	0.0326	0.1504	0.1094	0.1966	0.0383	0.0806	0.1071	0.0763	0.0751	0.0191	0.0159
P29	0.0858	0.0331	0.1677	0.1119	0.1992	0.0390	0.0689	0.1108	0.0825	0.0744	0.0145	0.0122
P30	0.0975	0.0359	0.1889	0.1393	0.1964	0.0380	0.0141	0.1090	0.0870	0.0615	0.0166	0.0158
P31	0.0552	0.0354	0.1929	0.1415	0.1957	0.0144	0.0810	0.0845	0.0789	0.0932	0.0156	0.0117
P32	0.0535	0.0459	0.1755	0.1157	0.2142	0.0387	0.0519	0.1227	0.0782	0.0708	0.0135	0.0194
P33	0.0722	0.0290	0.1922	0.1242	0.1979	0.0362	0.0551	0.0877	0.0782	0.0960	0.0188	0.0125
P34	0.0665	0.0325	0.1498	0.1447	0.1893	0.0369	0.1257	0.0900	0.0626	0.0755	0.0164	0.0101
P35	0.0593	0.0449	0.1575	0.1373	0.1943	0.0378	0.0863	0.1110	0.0793	0.0615	0.0167	0.0141
P36	0.0623	0.0408	0.1869	0.1284	0.1839	0.0355	0.0708	0.0856	0.0926	0.0822	0.0147	0.0163
P37	0.0547	0.0350	0.1616	0.1438	0.1993	0.0361	0.0510	0.1062	0.0762	0.0985	0.0184	0.0192

	P38	0.0881	0.0481	0.1638	0.1260	0.1999	0.0263	0.0685	0.0837	0.0947	0.0652	0.0196	0.0161
	P39	0.0661	0.0261	0.1667	0.1360	0.1964	0.0388	0.0755	0.0970	0.0910	0.0806	0.0101	0.0157
	P40	0.0551	0.0299	0.1701	0.1407	0.1965	0.0372	0.1052	0.0844	0.0680	0.0740	0.0200	0.0189
	P41	0.0783	0.0349	0.1429	0.1464	0.1888	0.0390	0.0614	0.1139	0.0640	0.0935	0.0182	0.0187
	P42	0.0594	0.0226	0.1709	0.1493	0.1892	0.0389	0.0872	0.0949	0.0732	0.0803	0.0197	0.0144
	P43	0.0576	0.0488	0.1647	0.1232	0.1972	0.0378	0.0755	0.1102	0.0687	0.0831	0.0179	0.0153
	P44	0.0940	0.0419	0.1612	0.0990	0.1978	0.0357	0.0946	0.1063	0.0651	0.0752	0.0106	0.0186
	P45	0.0622	0.0288	0.1772	0.1256	0.1972	0.0380	0.0815	0.0892	0.0701	0.0989	0.0176	0.0137
	P46	0.0647	0.0344	0.1873	0.1072	0.1978	0.0390	0.0481	0.1196	0.1000	0.0727	0.0102	0.0190
	P47	0.0831	0.0276	0.1572	0.1293	0.1937	0.0381	0.0784	0.1054	0.0798	0.0745	0.0167	0.0162
	P48	0.0852	0.0355	0.1712	0.1259	0.1978	0.0386	0.0598	0.0980	0.0891	0.0720	0.0164	0.0105
	P01	0.0809	0.0328	0.1326	0.1448	0.1992	0.0387	0.0797	0.0848	0.0826	0.0896	0.0183	0.0160
	P02	0.0592	0.0499	0.1755	0.1379	0.1926	0.0387	0.0531	0.1099	0.0798	0.0746	0.0151	0.0137
	P03	0.0843	0.0253	0.1468	0.1420	0.1968	0.0382	0.0531	0.1213	0.0827	0.0874	0.0105	0.0116
	P04	0.0732	0.0245	0.1745	0.1307	0.1953	0.0322	0.0936	0.0952	0.0707	0.0726	0.0200	0.0175
	P05	0.0711	0.0227	0.1654	0.1440	0.1960	0.0377	0.0471	0.1247	0.0976	0.0707	0.0130	0.0100
	P06	0.0781	0.0267	0.1646	0.1430	0.1954	0.0336	0.0661	0.1003	0.0802	0.0818	0.0176	0.0126
	P07	0.0782	0.0408	0.1395	0.1372	0.1996	0.0344	0.0831	0.1173	0.0669	0.0747	0.0176	0.0107
	P08	0.0712	0.0447	0.1783	0.1207	0.1867	0.0344	0.0689	0.0882	0.0795	0.0937	0.0174	0.0163
	P09	0.0895	0.0412	0.1648	0.1464	0.1636	0.0235	0.0718	0.1057	0.0794	0.0813	0.0148	0.0180
	P10	0.0676	0.0370	0.1919	0.1491	0.1693	0.0257	0.0804	0.0890	0.0738	0.0811	0.0163	0.0188
34290408	P11	0.0938	0.0410	0.1344	0.1370	0.1959	0.0359	0.0366	0.1210	0.0898	0.0822	0.0156	0.0168
	P12	0.0993	0.0218	0.1429	0.1388	0.1956	0.0318	0.0800	0.1142	0.0648	0.0757	0.0151	0.0200
	P13	0.0945	0.0279	0.1376	0.1420	0.1980	0.0323	0.0558	0.1186	0.0615	0.0967	0.0188	0.0163
	P14	0.0936	0.0473	0.1689	0.1126	0.1996	0.0319	0.0071	0.1236	0.0912	0.0933	0.0124	0.0185
	P15	0.0823	0.0347	0.1532	0.1244	0.1981	0.0366	0.0818	0.1211	0.0695	0.0664	0.0127	0.0192
	P16	0.0666	0.0228	0.1656	0.1441	0.1956	0.0370	0.0695	0.0915	0.0937	0.0795	0.0200	0.0141
	P17	0.0540	0.0395	0.1711	0.1381	0.1909	0.0368	0.1038	0.0983	0.0611	0.0811	0.0113	0.0140
	P18	0.0960	0.0438	0.1594	0.1467	0.1580	0.0269	0.0564	0.1147	0.0967	0.0651	0.0170	0.0193
	P19	0.0853	0.0328	0.1767	0.1261	0.1792	0.0338	0.1050	0.1069	0.0611	0.0644	0.0168	0.0119
	P20	0.0858	0.0351	0.1322	0.1470	0.1934	0.0355	0.0463	0.1231	0.0904	0.0822	0.0142	0.0148
	P21	0.0825	0.0297	0.1935	0.1099	0.1814	0.0332	0.0258	0.1166	0.0988	0.0968	0.0124	0.0194
	P22	0.0796	0.0386	0.1434	0.1307	0.1986	0.0386	0.0172	0.1282	0.0924	0.0979	0.0166	0.0182

P23	0.0771	0.0456	0.1579	0.1178	0.1948	0.0388	0.0635	0.1061	0.0925	0.0779	0.0105	0.0175
P24	0.0993	0.0238	0.1444	0.1340	0.1995	0.0366	0.0509	0.1213	0.0646	0.0894	0.0165	0.0197
P25	0.0507	0.0335	0.1832	0.1289	0.1995	0.0355	0.0634	0.0944	0.0847	0.0958	0.0194	0.0110
P26	0.0555	0.0319	0.1767	0.1265	0.1998	0.0390	0.0723	0.1010	0.0984	0.0778	0.0101	0.0110
P27	0.0832	0.0409	0.1419	0.1311	0.1934	0.0385	0.0787	0.1173	0.0649	0.0865	0.0120	0.0116
P28	0.0698	0.0432	0.1679	0.1434	0.1933	0.0328	0.0812	0.0996	0.0635	0.0724	0.0132	0.0197
P29	0.0815	0.0408	0.1586	0.1107	0.1995	0.0382	0.1116	0.1066	0.0601	0.0620	0.0128	0.0176
P30	0.0778	0.0383	0.1362	0.1427	0.1967	0.0379	0.0728	0.0903	0.0829	0.0941	0.0190	0.0113
P31	0.0902	0.0370	0.1549	0.1143	0.1962	0.0366	0.0894	0.1211	0.0712	0.0610	0.0160	0.0121
P32	0.0903	0.0201	0.1583	0.1287	0.1942	0.0382	0.0961	0.0853	0.0942	0.0613	0.0138	0.0195
P33	0.0904	0.0321	0.1902	0.1104	0.1677	0.0384	0.0604	0.1167	0.0877	0.0813	0.0138	0.0109
P34	0.0743	0.0429	0.1670	0.1320	0.1793	0.0363	0.0795	0.1125	0.0780	0.0716	0.0139	0.0127
P35	0.0531	0.0445	0.1835	0.1307	0.1957	0.0242	0.1059	0.1063	0.0649	0.0621	0.0149	0.0142
P36	0.0533	0.0264	0.1743	0.1500	0.1943	0.0355	0.0701	0.1265	0.0712	0.0708	0.0166	0.0110
P37	0.0550	0.0258	0.1927	0.1498	0.1949	0.0218	0.0644	0.1084	0.0661	0.0884	0.0153	0.0174
P38	0.0923	0.0432	0.1708	0.1262	0.1948	0.0065	0.0372	0.1290	0.0755	0.0849	0.0200	0.0196
P39	0.0988	0.0278	0.1785	0.0917	0.1943	0.0380	0.0540	0.1165	0.0960	0.0656	0.0199	0.0189
P40	0.0800	0.0487	0.1875	0.1075	0.1725	0.0354	0.1002	0.1032	0.0654	0.0736	0.0139	0.0121
P41	0.0995	0.0347	0.1914	0.1019	0.1805	0.0236	0.0646	0.0908	0.0841	0.0997	0.0117	0.0175
P42	0.0933	0.0255	0.1658	0.1486	0.1852	0.0294	0.0630	0.1194	0.0820	0.0620	0.0102	0.0156
P43	0.0811	0.0336	0.1329	0.1447	0.1985	0.0386	0.0623	0.1169	0.0852	0.0690	0.0194	0.0178
P44	0.0535	0.0272	0.1972	0.1445	0.1691	0.0377	0.0394	0.1205	0.0866	0.0868	0.0200	0.0175
P45	0.0674	0.0280	0.1824	0.1281	0.1924	0.0379	0.0632	0.1239	0.0707	0.0776	0.0183	0.0101
P46	0.0639	0.0426	0.1769	0.1314	0.1907	0.0348	0.0713	0.1021	0.0649	0.0841	0.0200	0.0173
P47	0.0676	0.0420	0.1417	0.1423	0.1995	0.0378	0.0998	0.0841	0.0841	0.0698	0.0113	0.0200
P48	0.0654	0.0308	0.1578	0.1425	0.1972	0.0373	0.0408	0.1154	0.0956	0.0780	0.0194	0.0198
P49	0.0694	0.0472	0.1535	0.1455	0.1925	0.0227	0.0753	0.1242	0.0664	0.0729	0.0193	0.0111
P50	0.0971	0.0239	0.1620	0.1364	0.1925	0.0317	0.0771	0.1008	0.0778	0.0679	0.0171	0.0157
P51	0.0707	0.0210	0.1815	0.1255	0.1992	0.0369	0.0996	0.0895	0.0646	0.0889	0.0108	0.0118
P52	0.0624	0.0423	0.1956	0.1307	0.1858	0.0162	0.0843	0.0843	0.0741	0.0917	0.0139	0.0187
P53	0.0539	0.0461	0.1516	0.1451	0.1998	0.0349	0.0759	0.0987	0.0827	0.0801	0.0164	0.0148
P54	0.0620	0.0252	0.1602	0.1458	0.1980	0.0380	0.0671	0.1086	0.0798	0.0872	0.0151	0.0130
P55	0.0886	0.0488	0.1893	0.1159	0.1848	0.0305	0.0490	0.1239	0.0647	0.0710	0.0140	0.0195

	P56	0.0740	0.0426	0.1946	0.1098	0.1830	0.0264	0.0758	0.0986	0.0786	0.0806	0.0175	0.0185
	P57	0.0519	0.0229	0.1840	0.1385	0.1936	0.0382	0.0807	0.1139	0.0807	0.0621	0.0146	0.0189
	P01	0.0809	0.0443	0.1813	0.0989	0.1861	0.0389	0.0701	0.1109	0.0780	0.0805	0.0161	0.0140
	P02	0.0862	0.0424	0.1749	0.1401	0.1767	0.0168	0.0742	0.1043	0.0829	0.0638	0.0186	0.0191
	P03	0.0847	0.0226	0.1973	0.1196	0.1691	0.0378	0.0344	0.1260	0.0794	0.0993	0.0197	0.0101
	P04	0.0541	0.0498	0.1505	0.1445	0.1950	0.0376	0.0688	0.1138	0.0932	0.0694	0.0125	0.0108
	P05	0.0916	0.0265	0.1986	0.1452	0.1924	0.0374	0.0073	0.1295	0.0722	0.0709	0.0106	0.0178
	P06	0.0760	0.0385	0.1318	0.1483	0.1973	0.0376	0.0909	0.0868	0.0836	0.0791	0.0131	0.0170
	P07	0.0579	0.0239	0.1733	0.1374	0.1991	0.0390	0.0603	0.1138	0.0872	0.0781	0.0158	0.0142
	P08	0.0592	0.0284	0.1893	0.1355	0.1787	0.0385	0.0650	0.0827	0.0983	0.0921	0.0140	0.0183
	P09	0.0885	0.0429	0.1539	0.1320	0.1849	0.0307	0.0863	0.0877	0.0936	0.0729	0.0103	0.0163
	P10	0.0736	0.0344	0.1711	0.1366	0.1957	0.0217	0.0414	0.1136	0.0957	0.0869	0.0177	0.0116
	P11	0.0828	0.0446	0.1680	0.1051	0.1986	0.0365	0.0719	0.0983	0.0636	0.0985	0.0167	0.0154
	P12	0.0963	0.0462	0.1450	0.1450	0.1798	0.0190	0.0584	0.1117	0.0929	0.0729	0.0183	0.0145
	P13	0.0702	0.0457	0.1682	0.1179	0.1929	0.0346	0.1034	0.1090	0.0630	0.0645	0.0119	0.0187
	P14	0.0905	0.0274	0.1440	0.1290	0.1995	0.0389	0.0730	0.1082	0.0958	0.0662	0.0126	0.0149
	P15	0.0631	0.0310	0.1612	0.1487	0.1995	0.0363	0.0754	0.0947	0.0994	0.0605	0.0173	0.0129
33958794	P16	0.0524	0.0322	0.1768	0.1449	0.1996	0.0348	0.0535	0.1152	0.0635	0.0927	0.0179	0.0165
	P17	0.0649	0.0419	0.1333	0.1500	0.1999	0.0390	0.0235	0.1280	0.0867	0.0964	0.0168	0.0196
	P18	0.0947	0.0440	0.1344	0.1494	0.1873	0.0287	0.0356	0.1155	0.0787	0.0968	0.0150	0.0199
	P19	0.0875	0.0253	0.1895	0.1217	0.1728	0.0327	0.0172	0.1243	0.0930	0.0980	0.0193	0.0187
	P20	0.0823	0.0293	0.1438	0.1362	0.1998	0.0376	0.0904	0.0964	0.0804	0.0702	0.0161	0.0175
	P21	0.0977	0.0358	0.1827	0.1145	0.1871	0.0354	0.0722	0.0913	0.0873	0.0699	0.0138	0.0123
	P22	0.0943	0.0369	0.1579	0.1072	0.1984	0.0370	0.0941	0.0972	0.0619	0.0760	0.0193	0.0198
	P23	0.0754	0.0454	0.1676	0.1489	0.1935	0.0247	0.0356	0.0889	0.0983	0.0908	0.0159	0.0150
	P24	0.0779	0.0350	0.1628	0.1195	0.1953	0.0387	0.1127	0.0909	0.0647	0.0775	0.0135	0.0115
	P25	0.0853	0.0427	0.1659	0.1418	0.1564	0.0380	0.0905	0.0951	0.0798	0.0732	0.0198	0.0115
	P26	0.0843	0.0407	0.1672	0.1141	0.1844	0.0385	0.0511	0.1211	0.0968	0.0682	0.0148	0.0188
	P27	0.0620	0.0387	0.1763	0.1326	0.1949	0.0253	0.1113	0.0958	0.0763	0.0625	0.0130	0.0113
	P28	0.0890	0.0239	0.1887	0.1024	0.1995	0.0348	0.0164	0.1228	0.0955	0.0987	0.0176	0.0107
	P29	0.0513	0.0295	0.1847	0.1443	0.1945	0.0277	0.0589	0.1252	0.0838	0.0687	0.0117	0.0197
	P30	0.0602	0.0262	0.1605	0.1440	0.1998	0.0385	0.0977	0.0849	0.0951	0.0728	0.0103	0.0100
	P31	0.0781	0.0477	0.1597	0.1198	0.1887	0.0583	0.0559	0.0965	0.0952	0.0661	0.0199	0.0141

P32	0.0905	0.0271	0.1542	0.1434	0.1754	0.0386	0.0908	0.0959	0.0797	0.0747	0.0100	0.0197
P33	0.0533	0.0358	0.1712	0.1480	0.1939	0.0335	0.0906	0.0807	0.0768	0.0849	0.0134	0.0179
P34	0.0564	0.0362	0.1549	0.1482	0.1972	0.0382	0.0796	0.0894	0.0996	0.0714	0.0136	0.0153
P35	0.0893	0.0266	0.1743	0.1255	0.1771	0.0388	0.0993	0.0947	0.0682	0.0729	0.0181	0.0152
P36	0.0978	0.0315	0.1654	0.1484	0.1958	0.0046	0.0548	0.0893	0.0989	0.0851	0.0182	0.0102
P37	0.0814	0.0473	0.1395	0.1475	0.1829	0.0379	0.0960	0.0937	0.0639	0.0805	0.0184	0.0110
P38	0.0931	0.0488	0.1342	0.1153	0.1987	0.0389	0.1198	0.0923	0.0677	0.0662	0.0100	0.0150
P39	0.0828	0.0354	0.1794	0.1174	0.1956	0.0289	0.0746	0.1210	0.0669	0.0695	0.0184	0.0101
P40	0.0580	0.0425	0.1820	0.1322	0.1871	0.0298	0.1101	0.0817	0.0724	0.0738	0.0193	0.0111
P41	0.0999	0.0404	0.1433	0.1453	0.1800	0.0332	0.0731	0.1147	0.0627	0.0783	0.0136	0.0155
P42	0.0730	0.0326	0.1488	0.1380	0.1977	0.0413	0.0880	0.0928	0.0643	0.0957	0.0109	0.0169
P43	0.0597	0.0281	0.1777	0.1484	0.1888	0.0341	0.0803	0.0958	0.0802	0.0842	0.0121	0.0106
P44	0.0610	0.0498	0.1661	0.1155	0.1977	0.0447	0.0678	0.0923	0.0789	0.0970	0.0116	0.0176
P45	0.0568	0.0335	0.1869	0.1315	0.1916	0.0333	0.0536	0.1235	0.0641	0.0944	0.0178	0.0130
P46	0.0537	0.0397	0.1982	0.1445	0.1930	0.0280	0.0829	0.0823	0.0711	0.0726	0.0183	0.0157
P47	0.0906	0.0373	0.1871	0.1124	0.1699	0.0342	0.0366	0.1231	0.0836	0.0980	0.0127	0.0145
P48	0.0519	0.0270	0.1976	0.1305	0.1951	0.0335	0.0517	0.1207	0.0661	0.0900	0.0162	0.0197
P49	0.0976	0.0363	0.1466	0.1296	0.1956	0.0309	0.0643	0.1122	0.0938	0.0627	0.0190	0.0114
P50	0.0743	0.0208	0.1826	0.1292	0.1859	0.0375	0.0894	0.0910	0.0667	0.0902	0.0167	0.0157
P51	0.0869	0.0226	0.1902	0.1394	0.1596	0.0370	0.0840	0.0979	0.0725	0.0824	0.0117	0.0158
P52	0.0509	0.0425	0.1787	0.1393	0.1824	0.0378	0.0920	0.0827	0.0749	0.0948	0.0117	0.0123
P53	0.0765	0.0306	0.1903	0.1258	0.1912	0.0280	0.0427	0.0958	0.0947	0.0877	0.0192	0.0175
P54	0.0846	0.0335	0.1924	0.1290	0.1603	0.0323	0.0711	0.0842	0.0854	0.0935	0.0180	0.0157
P55	0.0860	0.0306	0.1800	0.1258	0.1921	0.0234	0.0654	0.1259	0.0609	0.0745	0.0165	0.0189
P56	0.0919	0.0376	0.1449	0.1304	0.1862	0.0384	0.0954	0.0986	0.0673	0.0806	0.0147	0.0140
P57	0.0603	0.0475	0.1599	0.1362	0.1896	0.0361	0.0564	0.1268	0.0921	0.0644	0.0120	0.0187
P58	0.0826	0.0319	0.1353	0.1414	0.1994	0.0386	0.0812	0.1271	0.0711	0.0645	0.0133	0.0136
P59	0.0708	0.0443	0.1788	0.1165	0.1989	0.0270	0.0661	0.1159	0.0738	0.0783	0.0164	0.0132
P60	0.0967	0.0289	0.1727	0.1097	0.1829	0.0387	0.0552	0.1098	0.0872	0.0853	0.0141	0.0188
P61	0.0652	0.0490	0.1917	0.0950	0.1933	0.0366	0.0912	0.0837	0.0766	0.0863	0.0141	0.0173
P62	0.0935	0.0458	0.1779	0.1007	0.1819	0.0306	0.0944	0.0889	0.0818	0.0719	0.0131	0.0195
P63	0.0877	0.0372	0.1602	0.1339	0.1808	0.0314	0.0764	0.1128	0.0897	0.0600	0.0136	0.0163
P64	0.0705	0.0248	0.1636	0.1346	0.1995	0.0370	0.0783	0.1130	0.0699	0.0789	0.0164	0.0135

P65	0.0885	0.0336	0.1637	0.1196	0.1995	0.0387	0.0565	0.0992	0.0994	0.0663	0.0189	0.0161
P66	0.0738	0.0357	0.1468	0.1440	0.1984	0.0367	0.0988	0.0874	0.0636	0.0926	0.0119	0.0103
P67	0.0612	0.0268	0.1764	0.1295	0.1990	0.0384	0.0850	0.1064	0.0718	0.0729	0.0131	0.0195
P68	0.0887	0.0475	0.1980	0.0923	0.1761	0.0351	0.1094	0.0805	0.0672	0.0801	0.0121	0.0130
P69	0.0969	0.0488	0.1414	0.1060	0.1981	0.0387	0.0988	0.0976	0.0737	0.0680	0.0137	0.0183
P70	0.0848	0.0305	0.1897	0.1350	0.1546	0.0376	0.0708	0.0859	0.0960	0.0862	0.0139	0.0150
P71	0.0733	0.0488	0.1359	0.1463	0.1975	0.0358	0.0500	0.1277	0.0820	0.0650	0.0188	0.0189
P72	0.0796	0.0237	0.1413	0.1481	0.1984	0.0390	0.0774	0.0970	0.0949	0.0728	0.0178	0.0100
P73	0.0709	0.0292	0.1537	0.1469	0.1940	0.0367	0.0809	0.0912	0.0921	0.0724	0.0164	0.0156
P74	0.0834	0.0378	0.1421	0.1312	0.1989	0.0381	0.0786	0.1072	0.0802	0.0747	0.0108	0.0170
P75	0.0737	0.0478	0.1734	0.1495	0.1694	0.0362	0.0672	0.0933	0.0624	0.0997	0.0137	0.0137
P76	0.0596	0.0257	0.1563	0.1497	0.1996	0.0381	0.0681	0.1029	0.0785	0.0944	0.0151	0.0120
P77	0.0806	0.0224	0.1620	0.1439	0.1822	0.0385	0.0780	0.1300	0.0705	0.0622	0.0117	0.0180
P78	0.0619	0.0494	0.1626	0.1456	0.1869	0.0286	0.0897	0.0988	0.0606	0.0816	0.0182	0.0161
P79	0.0640	0.0231	0.1814	0.1304	0.1991	0.0364	0.1004	0.0828	0.0661	0.0901	0.0144	0.0118
P80	0.0802	0.0468	0.1616	0.1330	0.1783	0.0319	0.0721	0.1142	0.0855	0.0643	0.0185	0.0136
